# Dynamics of humoral and cellular response to three doses of anti-SARS-CoV-2 BNT162b2 vaccine in patients with hematological malignancies and older subjects

**DOI:** 10.3389/fimmu.2023.1221587

**Published:** 2024-01-26

**Authors:** Valentina Laquintana, Carla Mottini, Francesco Marchesi, Benedetta Marcozzi, Irene Terrenato, Eleonora Sperandio, Luisa de Latouliere, Francesca Carrieri, Fulvia Pimpinelli, Martina Pontone, Raul Pellini, Flaminia Campo, Laura Conti, Celeste Accetta, Chiara Mandoj, Fabrizio Petrone, Ornella Di Bella, Branka Vujovic, Aldo Morrone, Mirco Compagnone, Eugenia Principato, Eleonora Pinto, Elena Papa, Paolo Falcucci, Antonia La Malfa, Matteo Pallocca, Federico De Marco, Giulia Piaggio, Gennaro Ciliberto, Andrea Mengarelli, Simona di Martino

**Affiliations:** ^1^ UOC Anatomy Pathology, Biobank IRCCS Regina Elena National Cancer Institute, Istituti Fisioterapici Ospitalieri (IFO), Rome, Italy; ^2^ UOSD SAFU, IRCCS Regina Elena National Cancer Institute, Istituti Fisioterapici Ospitalieri (IFO), Rome, Italy; ^3^ UOSD Hematology and Stem Cell Transplant Unit, IRCCS Regina Elena National Cancer Institute, Istituti Fisioterapici Ospitalieri (IFO), Rome, Italy; ^4^ UOSD Clinical Trial Center, Biostatistic and Bionformatic, IRCCS Regina Elena National Cancer Institute, Istituti Fisioterapici Ospitalieri (IFO), Rome, Italy; ^5^ UOC D.I.T.R.A.R. IRCCS Regina Elena National Cancer Institute, Istituti Fisioterapici Ospitalieri (IFO), Rome, Italy; ^6^ UOSD of Microbiology and Virology, IRCCS San Gallicano Dermatological Institute, Istituti Fisioterapici Ospitalieri (IFO), Rome, Italy; ^7^ UOC Otolaryngology Head and Neck Surgery, IRCCS Regina Elena National Cancer Institute, Istituti Fisioterapici Ospitalieri (IFO), Rome, Italy; ^8^ UOSD Clinical Pathology, IRCCS Regina Elena National Cancer Institute, Istituti Fisioterapici Ospitalieri (IFO), Rome, Italy; ^9^ Medical Direction, Istituti Fisioterapici Ospitalieri (IFO), Rome, Italy; ^10^ Scientific Direction, IRCCS San Gallicano Dermatological Institute, Istituti Fisioterapici Ospitalieri (IFO), Rome, Italy; ^11^ Neomatrix, Rome, Italy; ^12^ Università degli Studi “Magna Graecia” di Catanzaro, Catanzaro, Italy; ^13^ Takis srl, Rome, Italy; ^14^ Pharmacy Unit, IRCCS Regina Elena National Cancer Institute and San Gallicano Institute, Rome, Italy; ^15^ Scientific Direction, IRCCS Regina Elena National Cancer Institute, Istituti Fisioterapici Ospitalieri (IFO), Rome, Italy

**Keywords:** COVID-19, SARS-CoV-2, mRNA vaccine, humoral and cellular response, hematological patients

## Abstract

**Background:**

Few data are available about the durability of the response, the induction of neutralizing antibodies, and the cellular response upon the third dose of the anti-severe acute respiratory syndrome coronavirus 2 (SARS-CoV-2) vaccine in hemato-oncological patients.

**Objective:**

To investigate the antibody and cellular response to the BNT162b2 vaccine in patients with hematological malignancy.

**Methods:**

We measured SARS-CoV-2 anti-spike antibodies, anti-*Omicron* neutralizing antibodies, and T-cell responses 1 month after the third dose of vaccine in 93 fragile patients with hematological malignancy (FHM), 51 fragile not oncological subjects (FNO) aged 80–92, and 47 employees of the hospital (healthcare workers, (HW), aged 23-66 years. Blood samples were collected at day 0 (T0), 21 (T1), 35 (T2), 84 (T3), 168 (T4), 351 (T pre-3D), and 381 (T post-3D) after the first dose of vaccine. Serum IgG antibodies against S1/S2 antigens of SARS-CoV-2 spike protein were measured at every time point. Neutralizing antibodies were measured at T2, T3 (anti-Alpha), T4 (anti-Delta), and T post-3D (anti-*Omicron*). T cell response was assessed at T post-3D.

**Results:**

An increase in anti-S1/S2 antigen antibodies compared to T0 was observed in the three groups at T post-3D. After the third vaccine dose, the median antibody level of FHM subjects was higher than after the second dose and above the putative protection threshold, although lower than in the other groups. The neutralizing activity of antibodies against the *Omicron* variant of the virus was tested at T2 and T post-3D. 42.3% of FHM, 80,0% of FNO, and 90,0% of HW had anti-*Omicron* neutralizing antibodies at T post-3D. To get more insight into the breadth of antibody responses, we analyzed neutralizing capacity against BA.4/BA.5, BF.7, BQ.1, XBB.1.5 since also for the *Omicron* variants, different mutations have been reported especially for the spike protein. The memory T-cell response was lower in FHM than in FNO and HW cohorts. Data on breakthrough infections and deaths suggested that the positivity threshold of the test is protective after the third dose of the vaccine in all cohorts.

**Conclusion:**

FHM have a relevant response to the BNT162b2 vaccine, with increasing antibody levels after the third dose coupled with, although low, a T-cell response. FHM need repeated vaccine doses to attain a protective immunological response.

## Introduction

Real-world studies on the effectiveness of two vaccine doses in preventing severe acute respiratory syndrome coronavirus 2 (SARS-CoV-2) infection have shown that the BNT162b2 mRNA vaccine (Comirnaty, BioNTech Manufacturing GmbH [Germany], Pfizer Manufacturing Belgium NV [Belgium]) prevents severe COVID-19 disease in subjects aged over 12 years (1, 2). SARS-CoV-2 evolves mutations to escape vaccine-and infection-acquired immunity, raising concern about the potential impact of new variants on vaccination programs (3). The *Omicron* variant was first detected in November 2021 and was found to contain numerous mutations in the spike protein, resulting in enhanced transmissibility, partial escape from previously established neutralizing antibodies to wild-type SARS-CoV-2, and possible re-infection (4, 5). Several sub-lineages of the Omicron variant have emerged, all exhibiting partial escape from wild-type spike-based vaccine-elicited neutralizing antibodies (6). Data from clinical trials and real-world studies suggest that current vaccines retain effectiveness against the *Alpha* variant (B.1.1.7), although effectiveness may be reduced against the *Beta* variant (B.1.351) and the *Delta* variant (B.1.617.2); however, effectiveness against severe disease and hospitalization caused by *Delta* remains high. Booster programs using mRNA vaccines have been shown to restore protection against infection and symptomatic disease by the *Omicron* variant (3).

However, as the incidence of COVID-19 progressively increased after the second vaccination campaign, governments worldwide fostered a third dose of vaccine as a booster. Several studies evaluated the humoral, and in a few instances the cellular, response following the third dose of the BNT162b2 vaccine in healthy adult subjects and frail populations, including patients with hematological malignancies and older subjects (7–27). However, few data are available about the durability of the response, and the induction of neutralizing antibodies upon the third dose of vaccine in hemato-oncological patients (28–34) and in older subjects (35–39). Overall, in patients with hematological malignancy, repeated vaccination obtains seroconversion in most subjects and the more doses the higher the serological response (28, 30–34). Nevertheless, results are inconsistent on the possibility of having seroconversion with further doses in those who had not reached the positivity threshold with the second dose (31, 33). The neutralizing capacity against the *Omicron* variant seems poor (32). Finally, Firinu et al. found that patients treated with antiCD20 had higher IFN-γ response compared to patients with immune-mediated inflammatory disease, who were naïve to antiCD20 (29). It was reported that subjects >80 years old had lower humoral response than younger subjects to the second vaccine dose (36), but an increase in anti-spike antibodies after the third dose was observed both over 60 years and over 80 years (36, 38). The neutralizing activity against *Delta* and *Omicron* increases after the third dose in elders (35). Additionally, anti-spike IgG and neutralizing antibody levels remain adequate 3 months after the third BNT162b2 vaccine in healthy adults (39).

Indeed, whether the third dose of the BNT162b2 vaccine can reinforce anti-spike antibody levels, and the neutralizing power of antibodies and T-cell responses in fragile populations are still open questions. Such problems need to be addressed to guide future decisions about repeated anti-SARS-CoV-2 vaccinations in frail subjects.

To this aim, we compared the response to the third dose of the BNT162b2 vaccine and its efficacy in protecting against SARS-CoV-2 infection over time in fragile patients with hematological malignancy (FHM), fragile older not oncological subjects (FNO), and a cohort of subjects in good general health, healthcare workers (HW).

## Participants and methods

### Study design

A prospective observational study was carried out in the Istituti Fisioterapici Ospedalieri (IFO; Rome, Italy), a referral center for the treatment of oncological diseases in central Italy. During the COVID-19 pandemic, it was a vaccination center directed at its employees, oncological patients, and the general population over >80 years old.

The vaccination timing, with the BNT162b2 vaccine, started in December 2020 for employees of IFO and in March 2021 for oncological patients and the general population with the first dose of vaccine administration (T0), followed by the second dose after 21 days (T1) and ended with the third dose of the vaccine after 351 days (T pre-3D).

The study protocol complied with the Declaration of Helsinki and was approved by the institutional scientific ethics committees (IRCCS Lazio, protocol n.RS1446/20; protocol RS1463/21). All enrolled subjects released written informed consent for participation in the study.

### Participants

The study enrolled subjects who joined the vaccination campaign organized by IFO and received three doses of the vaccine. Three cohorts were recruited: a) patients aged >18 years, affected with a diagnosed hematological malignancy, at any stage, treated and followed-up at IFO (fragile hematological malignancy, FHM); b) subjects from the general population, aged over 80 years (fragile not oncological, FNO); c) employees of IFO, aged >18 years, in good general condition (healthcare workers, HW). Consecutive subjects of each group were included. All patients released informed consent to receive the vaccine doses and to participate in the study. FHM subjects receiving the first and the second dose in other vaccination centers were admitted, while all subjects had to receive the third dose in IFO. Data on the response to the first two doses in all these subjects have been previously published (40, 41).

### Time-course of analyses

**Figure 1:**
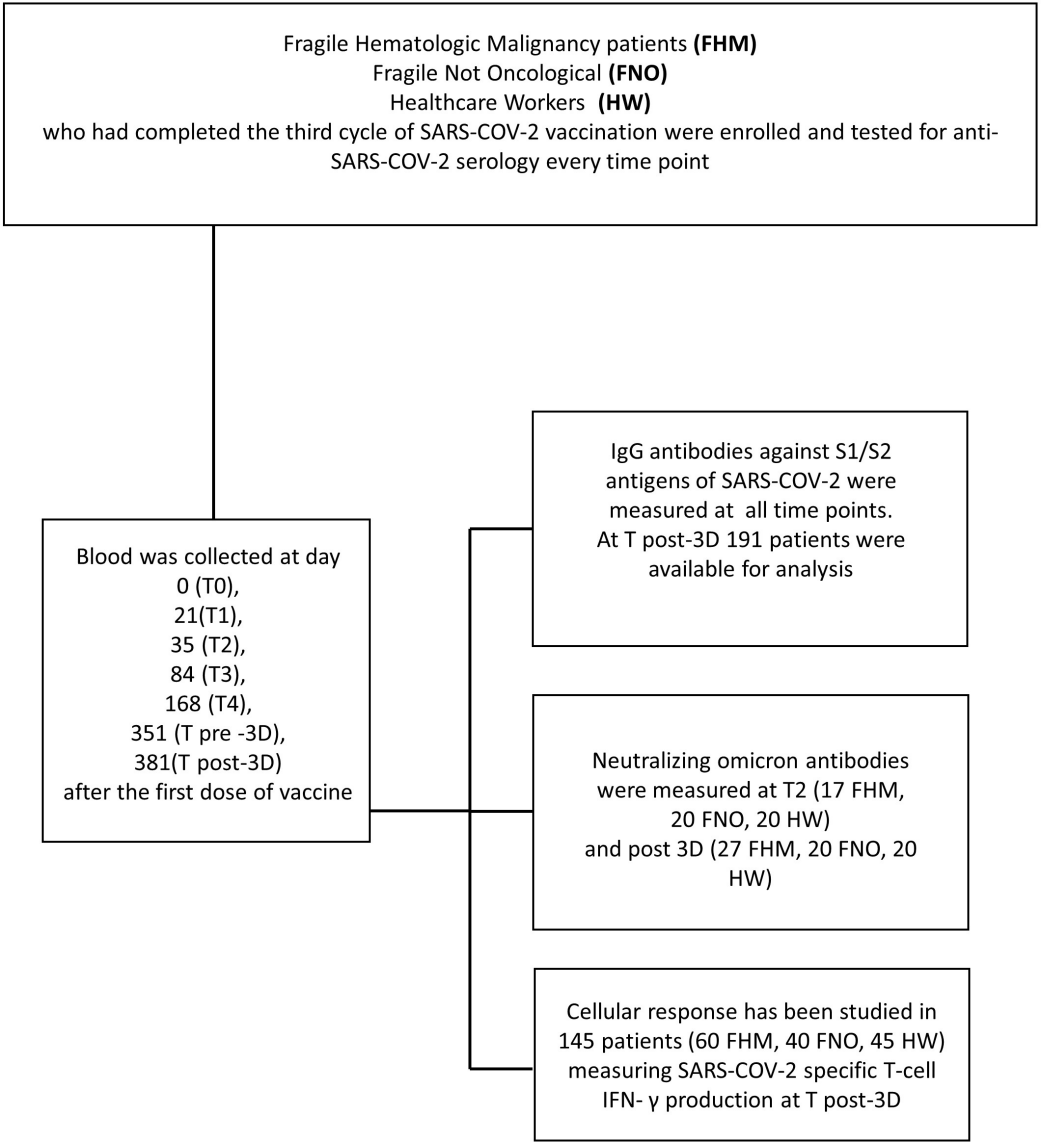
Consort flow diagram of the study. In the upper box, the three cohorts are indicated. The left box describes the time course of blood sample collection. In the right box executed procedures are listed.

Following the first dose of vaccination, the second dose of vaccine was administered exactly 21 days after the first one, and the third dose 351 days after the first one (**Figure 1**).

Blood samples were collected from each subject at day 0 (T0, the day of the first dose of vaccine administration), 21 (T1), 35 (T2), 84 (T3), 168 (T4), 351 (T pre-3Dose), and 381 (T post-3Dose) days after the first dose of vaccine (**Figure 1**).

**Table 1** and **Figure 1** report laboratory analyses performed at each time point. IgG antibodies against S1/S2 antigens of SARS-CoV-2 were assessed at each time point. Neutralizing anti-*Omicron* antibodies were assessed at T2 (35 days after the first vaccine dose) and at T post-3D (381 days after the first vaccine dose). Interferon-gamma (IFN-γ) secreted by T cells in response to SARS-CoV-2 antigens was measured in peripheral blood mononuclear cells (PBMCs) at T post-3D time point.

**Table 1:** Time course of assessments.

Assessments	T0	T1	T2	T3	T4	T pre- 3D	T post-3D
	Day of vaccine dose 1	The day of vaccine dose 2 21 days after dose 1	35 days after dose 1	84 days after dose 1	168 days after dose 1	The day of vaccine dose 3 351 days after dose 1	381 days after dose 1
**IgG antibodies against S1/S2 antigens of SARS-COV-2**	x	x	x	x	x	x	x
***Omicron* neutralizing antibodies**		x					x
**Cellular response**							x

### Serum isolation and serological studies

The serum isolation method is described in the **Supplementary Material**. The antibodies in the tested sera were quantified in arbitrary units per milliliter (AU/mL) with the LIAISON^®^ SARS-CoV-2 S1/S2 IgG and the LIAISON^®^ SARS-CoV-2 S1/S2 IgG TrimericS test kits. In all measurements in which the LIAISON^®^ SARS-CoV-2 TrimericS IgG assay (Emergency Use Authorization, EUA) was used, the AU/ml has been converted to BAU/mL according to the manufacturer’s information regarding the WHO standard. The conversion to BAU was AU/mL×2.6 (42, 43).

Binding Neutralization Assay Binding Neutralization tests are described in the **Supplementary Material**. Neutralizing anti-receptor-binding domain (RBD) antibodies against the A*lpha* variant of the virus were measured by the COVID-19 Spike-ACE2 Binding Assay Kit (Raybiotech Life, Inc., GA, USA). This test detects semi-quantitative antibodies against the SARS-CoV-2 spike protein in human serum. To test functional neutralizing antibodies against the *Delta* (B.1.617.2) and *Omicron* (B.1.1.529) variants of SARS-CoV-2, two commercially available ELISA kits were used: SARS-CoV-2 Neutralizing Antibodies Detection Kit (B.1.617.2 *Delta* variant), Cat. No. AG-48B-0007-KI01, Adipogen and SARS-CoV-2 Neutralizing Antibody Titer Serologic Assay Kit (B.1.1.529 *Omicron* variant), ACROBiosystems. Both assays identify the presence or absence of specific neutralizing antibodies by testing the interaction of the RBD of the SARS-CoV-2 spike protein and the recombinant human ACE2 receptor (hACE2). The Adipogen kit defines a cut-off value for neutralization of 20%, while for the ACROBiosystems kit, the cut-off value corresponds to 32%. The inhibition percentage of ≥20% or ≥32% indicates the presence of neutralizing antibodies against the SARS-CoV-2 *Delta* or *Omicron* variant, respectively. To test functional neutralizing antibodies against the *Omicron* variants (BA.4/BA.5/BF. 7/BQ.1/XBB.1.5) a commercial ELISA kit was used: Gene Tex SARS-CoV-2 Neutralizing Antibody, Cat. No. GTX538288. This is an *in vitro* assay for qualitative SARS-CoV-2 neutralizing antibody (Nab) screening. The inhibition percentage of ≥30% indicates the presence of neutralizing antibodies against the SARS-CoV-2 *Omicron* variants.

### Assessment of activated T effector cells

The methods used are described in the **Supplementary Material**.

PBMCs were isolated from “buffy coats” (whole blood concentrates without serum) via density gradient centrifugation using Lympholyte^®^-H Cell Separation Media (CEDARLANE^®^). The T-SPOT COVID-19 test was used to assess the activation of effector T cells. An enzyme-linked immunospot (ELISPOT) assay identifies T cells in PBMCs that release IFN-γ in response to stimulation with SARS-CoV-2 spike S1 and/or nucleocapsid peptides. It is a commercialized, standardized ELISPOT platform that allows reproducible measurements of T cells reactive to SARS-CoV-2 antigens. The T-SPOT COVID-19 test is built on the T-SPOT platform (Oxford Immunotec, a PerkinElmer Company). To quantify the activation of effector T cells, we used the T-SPOT COVID-19 test. As part of the T-cell response, SARS-CoV-2 antigens on the vaccine could activate both CD4+ and CD8+ effector T cells, which, if further stimulated by these antigens, produce IFN-γ cytokine. The spot-forming units (SFUs) were counted using an automated ELISPOT plate reader (CTL, Shaker Heights, OH, USA). Results were ‘invalid’ if the negative control had more than 10 SFUs or the positive control had fewer than 20 SFUs when the antigen wells were non-reactive. Results were ‘reactive’ when the SFUs in the higher of the two antigen wells minus the negative control were ≥6, ‘non-reactive’ when the SFUs in both antigen wells minus the negative control were ≤6, and ‘borderline’ when the SFUs in the higher of the antigen wells minus the negative control were 5, 6 or 7.

### *In vitro* stimulation of PBMCs

To test T cell activation, PBMCs were thawed and rested for 4 hours in 10% FBS-supplemented RPMI-1640. Then, 2×106 PBMCs were seeded in U-bottom 96-well plates in a total volume of 200 μl and stimulated for 12–16 hours at 37°C in 10% FBS-supplemented RPMI-1640 with PepTivator^®^ SARS-CoV-2 protein S peptide pool (Miltenyi Biotec, cat. 130-127-953) or CEFX Ultra SuperStim Pool (JPT, PM-CEFX-2) at the final concentration of 1 μg/mL of each peptide or with an equal volume of water, in the presence of Brefeldin A (Thermo Fisher Scientific, cat. 00-4506-51), as previously described (44).

### Flow cytometry staining and acquisition

Cells were stained for viability (LIVE/DEAD™ Fixable Violet Dead Cell Stain, Thermo Fisher Scientific, cat. L34955). Afterward, cells were incubated with Human Fc Block (BD, cat. 564220) followed by staining for surface markers using the following antibodies: CD3 (UCHT1) FITC, 1:200 (Thermo Fisher Scientific, cat. 11-0038-42), CD4 (RPA-T4) PerCP-eFluor 710, 1:100 (Thermo Fisher Scientific, cat. 46-0049-42), CCR7 (CD197) APC, 1:100 (BD Biosciences, cat. 566762), CD8 (SK1) APC-eFluor 780, 1:200 (Thermo Fisher Scientific, cat. 47-0087-42), CD45RA (HI100) BV605, 1:100 (BioLegend, cat. 304134) in FACS buffer for 20 minutes at 4°C. Afterward, samples were fixed and permeabilized using Cytofix/Cytoperm (BD Biosciences, cat. 554722) for 20 minutes at 4°C. Intracellular staining was performed in Perm/Wash buffer (BD Biosciences, cat. 554723) for 30 minutes at 4 °C with the following antibodies: IFNγ (4S.B3) PE, 1:100 (Thermo Fisher Scientific, cat. 12-7319-42), TNFα (MAb11) PC7, 1:100 (Thermo Fisher Scientific, cat. 25-7349-82). IFNγ and TNFα were assessed by intracellular cytokine staining (ICS) following stimulation overnight with the peptide pools, using an in-house validated flow cytometry antibody panel for Th1 response (45). The stained samples were acquired through a CytoFLEX flow cytometer (Beckman Coulter), and the data were analyzed using CytExpert software (Beckman Coulter). Cytokine expression in the presence of only water (with no peptides) was considered background and subtracted from the values measured with stimuli.

### Production of SARS-CoV-2 pseudoparticles

A neutralization assay was performed for the *Delta* and the *Omicron* variants, with pseudotyped particles on HEK293T-hACE2 cells transduced with SARS-CoV-2 pseudovirus previously incubated with a serial 2.5-fold dilution of heat-inactivated serum. The method is described in the **Supplementary Material**.

### Statistical analysis

Data were summarized through descriptive statistics.

Categorical data were synthetized through frequencies and percentage values while continuous data were reported through median values and the relative range (min-max). The correlation between variables was explored by Spearman’s Rho coefficient. Due to the nature of the distributions, the Kruskal-Wallis, Mann-Whitney, and Pearson’s Chi-square non-parametric tests with Bonferroni’s correction for multiple comparisons, when appropriate, were applied to assess potential differences among parameters. A p<0.05 was considered statistically significant. IBM SPSS v.21 and R v.4.2.0 were used for all statistical analyses.

In the analysis of IgG levels with the LIAISON^®^ SARS-CoV-2 S1/S2 IgG test kit we used as putatively protective, a 15 AU/mL cut-off as indicated by DiaSorin^®^. This putatively protective cut-off is considered valid to discriminate responders from non-responders to vaccination. It was identified based on a concordance level of more than 94.4% among the plaque reduction neutralization test, a gold standard for assessing the relative concentrations of virus-specific neutralizing antibodies. Moreover, a concordance of 100% at 80 AU/mL was demonstrated (42). In the analysis of IgG levels with the LIAISON^®^ SARS-CoV-2 TrimericS IgG test kit we used, as putatively protective, a 34 BAU/mL cut-off as indicated by DiaSorin^®^. Therefore, according to these cut-offs, we investigated the % of positive subjects before and after the third vaccine dose in the three cohorts.

## Results

### Study population and blood collection

**Table 2** reports the number of subjects enrolled in the study with evaluable data at each study time point. In total, 77 FHM, 77 FNO, and 62 HW had received the first dose of vaccine in our institution, IFO. At the second dose, we lost 9 FNO subjects. At the third dose, we enrolled 17 new FHM subjects who received doses 1 and 2 in other vaccine centers and we lost 17 FNO and 12 HW subjects who withdrew from the study.

**Table 2:** Number of subjects in the study at any time point with evaluable data.

Time points/days after vaccine dose 1	Fragile Hematological Malignancy (n)	Fragile, older, Not-Oncological (n)	Healthcare Workers (n)
**T0/0**	77	77	62
**T1/21**	77	68	62
**T2/35**	77	56	62
**T3/84**	77	54	62
**T4/168**	77	54	61
**T pre-3D/351**	94	60	50
**T post-3D/381**	93	51	47

**Table 3** reports the demographic characteristics of all enrolled subjects in the three cohorts and the clinical characteristics of FHM subjects. The mean age was 73 years for FHM, 81 years for FNO, and 48 years for the healthcare workers. Men were 63% of FHM, 49% of FNO, and 26% of HW. Among hematological malignancies, 31 (33%) were chronic lymphocytic leukemia (CLL)/small lymphocytic lymphoma, 34 (36%) myeloma, and 29 (31%) other myeloproliferative diseases. Among FHM, 39 patients were receiving monoclonal antibody therapies: 24 (25%) were on rituximab (anti-CD20) and 15 (16%) on daratumumab (anti-CD38) with an average time to start treatment of 3 and 5 months, respectively.

**Table 3:** Demographic characteristics of enrolled subjects and clinical characteristics of FHM, at T pre-3D.

Characteristics	Fragile Hematological Malignancy (n=94)	Fragile, older, Not-Oncological (n=60)	Healthcare Workers (n=50)
Age (years), median (IQR*)	73 (65 – 77)	81 (80 - 85)	48 (37 - 55)
Gender, n (%):			
• **Male**	59 (63)	29 (49)	11 (22)
• **Female**	35 (37)	31 (51)	39 (78)
Malignancy, n (%):			
• **Lymphoma chronic lymphocytic leukemia**	31(33)		
• **Malignant myeloma**	34 (36)		
• **Other myeloproliferative diseases**	29 (31)		
Active therapy, n (%):			
• **Anti-CD20 (rituximab)**	24 (25)		
• **Anti-CD38 (daratumumab)**	15 (16)		
• **No monoclonal anti-B-cell**	55 (59)		

*IQR, interquartile range.

### Dynamic of anti-SARS-CoV-2 IgG levels over time

**Figure 2:**
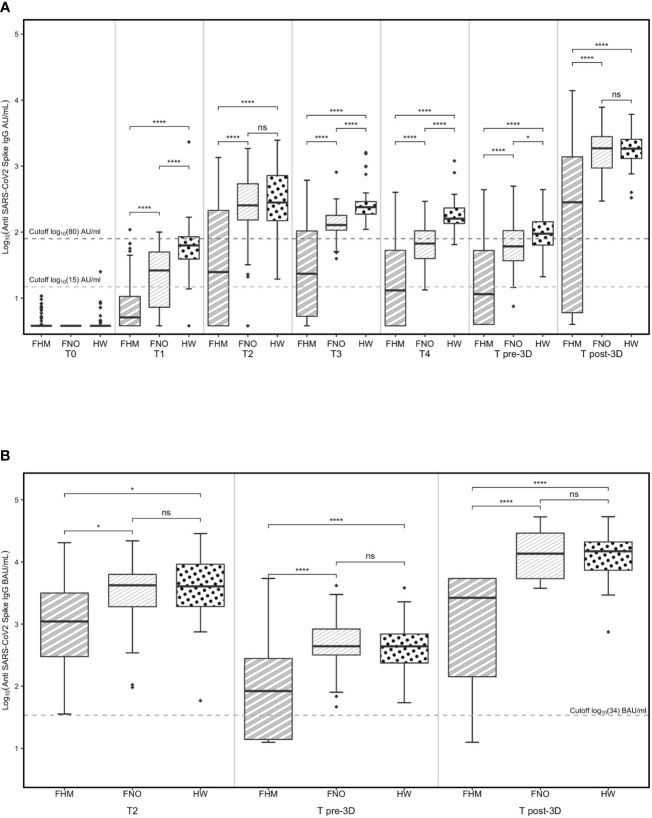
Levels of anti-SARS-CoV-2 IgG determined by **(A)** the LIAISON® SARS-CoV-2 S1/S2 IgG test kit and **(B)** the LIAISON® SARS-CoV-2 S1/S2 IgG TrimericS test kit. Levels of anti-SARS-CoV-2 IgG in fragile subjects with hematological malignancies (FHM), not oncologic (FNO) and healthcare worker (HW) cohorts over time **(A)**. Levels of anti-spike trimeric IgG in FHM, FNO and HW at specific time-points **(B)**. Statistical significances calculated by paired Wilcoxon test (comparisons through time-points) and by unpaired Mann-Whitney test (comparisons by groups) are reported in the graph. Diamonds represent outliers. *p<0.05, ****p<0.00001. ns, not significant.

Levels of anti-SARS-CoV-2 IgG in the three cohorts at every time point are shown in **Figure 2A**. The data are presented in AU/ml since they have been collected with the LIAISON^®^ SARS-CoV-2 S1/S2 IgG test kit that does not allow the conversion into BAU/ml.

At every time point, the median levels of IgG were lower in the cohort of FHM than in the other groups.

At the T2 time point, after 14 days from the second vaccination dose, the median levels of IgG were above the putatively protective cut-off of 15 AU/mL in the three cohorts (and progressively decreased until the third vaccination, the T pre-3D time point. Of note, the median IgG levels from T2 to T pre-3D remained below 15 AU/mL in FHM at T4 and T pre-3D time points, increasing after the third dose of vaccine (T post-3D). In the other two groups, although the IgG levels decreased at T3, T4, and T pre-3D, the putatively protective cut-off at 15 AU/mL was always reached.

The more stringent putatively protective level of IgG (cut-off at 80 AU/mL) was reached after the second dose of vaccine in FNO and HW; the median levels progressively decreased in both groups until the third dose of vaccine (T pre-3D). Both FNO and HW subjects had antibody levels above this cut-off after the third dose of vaccine (T post-3D). In FHM, the median levels of IgG reached this cut-off only after the third dose of vaccine (T post-3D).

The proportions of subjects with IgG levels above the 15 AU/mL cut-off were between 90% and 100% before and after the third dose of vaccine in FNO and HW subjects and were 44% before and 70% after the third dose in FHM (**Supplementary Figure 1A**).

The proportions of subjects with IgG levels above the 80 AU/mL cut-off were 12% in FHM, 35% in FNO, and 60% in HW subjects before the third dose of vaccine (**Supplementary Figure 1B**), and raised to 65% in FHM and to 98% in the two other groups after the third dose (**Supplementary Figure 1B**).

Between administering the second and the third dose of the vaccine, a second-generation test using the entire spike S1/S2 protein in its trimeric form was adopted for antibody measurement at our institute. To test whether the results obtained with the LIAISON^®^ SARS-CoV-2 S1/S2 IgG test kit were comparable to those obtained with the new test, samples at T2, T pre-3D, and T post-3D were also analyzed with the latter test. **Figure 2B** shows that the results are largely comparable to those in **Figure 2A**, obtained with the LIAISON^®^ SARS-CoV-2 S1/S2 IgG test kit. The results of this test further confirm that the vaccine response of FHM patients is much lower than that observed in the other two cohorts. The median levels of IgG anti-trimeric Spike were above the putatively protective cut-off indicated for this kit (34 BAU/mL) in the three cohorts of subjects at T2 but were significantly lower in the FHM cohort than in the other two groups. Both FNO and HW subjects had antibody levels above this cut-off before and after the third dose of the vaccine. In FHM, the median levels of IgG reached this cut-off only after the third dose of vaccine (T post-3D). However, 12 FHM subjects did not reach this cut-off even after the third dose of the vaccine. Among these subjects, 10 had lymphoma/CLL and had been on treatment with rituximab for a median time of 3 months, while one patient had myeloma and had been on treatment with daratumumab for 5 months (**Table 4**). Among the FHM subjects that reached the protective cut-off, only 2 out of 27 myeloma patients were treated with daratumumab. Conversely, 1 out of 38 patients treated with drugs not targeting B cells were non-responders. The median concentrations of IgGs are shown in **Table 5**.

**Table 4:** Antibody response to the third dose of the anti-SARS-CoV-2 vaccine evaluated on a subgroup of patients with hematological malignancy, according to the type of malignancy and monoclonal treatment.

Cut-off > 34 BAU/mL T post-3D				
	Lymphoma/CLL	Myeloma	Myeloproliferative neoplasms	p-value
**Not responders**	11	1	0	<0.001
**Responders**	2	27	21	
**Total**	13	28	21	
	Monoclonal Anti-CD20 (Rituximab)	Monoclonal Anti-CD38 (Daratumumab)	No monoclonal anti-B cells	p-value
**Not responders**	10	1	1	<0.001
**Responders**	0	12	38	
**Total**	10	13	39	

**Table 5:** Median concentrations of IgG in each group of subjects before (T pre-3D) and after (T post-3D) the third dose of vaccine.

T pre-3D			T post-3D		p-value
Group	IgG (BAU/mL)	min - max	IgG (BAU/mL)	min - max	
**FHM**	29.9	10.4 - 1141.4	735.8	10.4 - 36140.0	<0.001
**FNO**	161.2	20.8 - 1292.2	4836.0	767.0 - 20254.0	<0.001
**HW**	246.6	54.9 - 1141.4	4862.0	860.6 - 15834.0	<0.001

No significant differences across all time points were observed according to the gender of the subjects in the three cohorts (**Supplementary Figure 2**).

### Serum-neutralizing activity against the *Omicron* variants after the third vaccine dose

**Figure 3:**
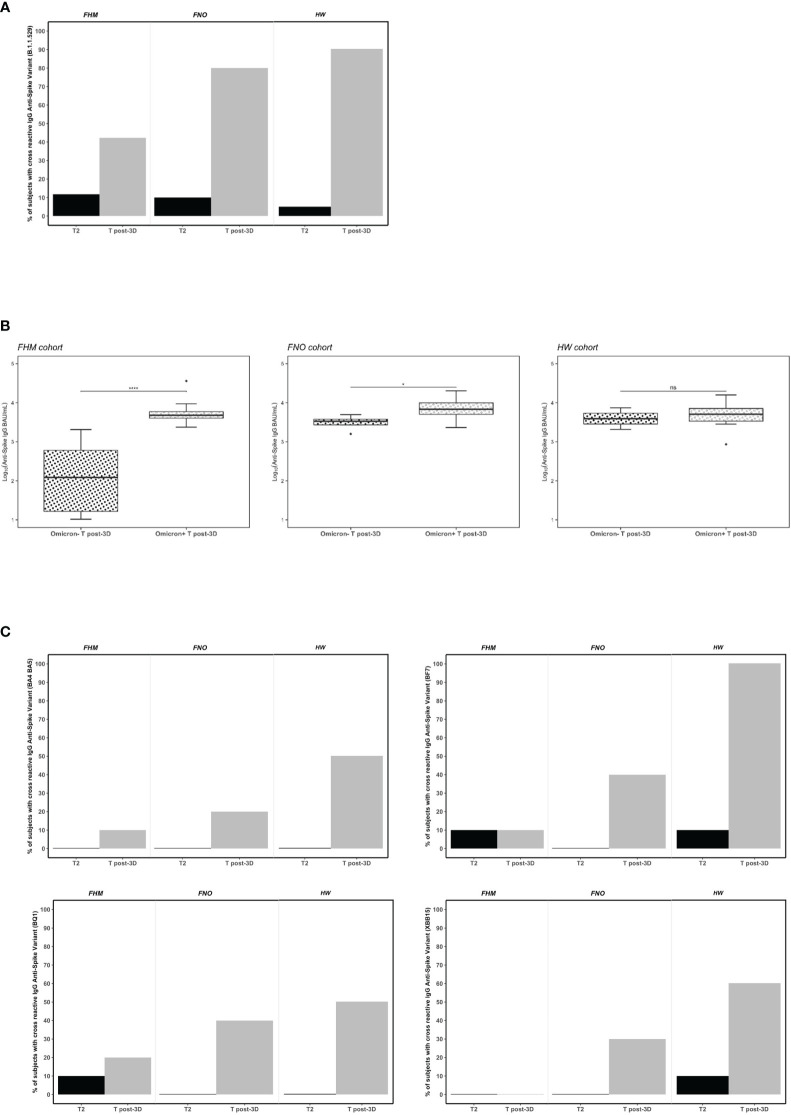
Serum neutralizing activity against the *Omicron* spike variants in fragile hematological malignancies (FHM), fragile not-oncologic subjects (FNO) and healthcare workers (HW) after the first dose (T2) and after the third dose (381 days from dose 1) of Pfizer BNT162b2 vaccine. **(A)** Data plotted are percentage values of positive subjects with cross-reactive IgG anti-spike variant (B.1.1529). The cut-off for serum neutralization is an inhibitory dilution used as recommended by the manufacturer. Comparisons among groups were conducted by Pearson’s Chi-square test at T2 and at T post-3D (p=ns and p<0.001, respectively). **(B)** Association of anti-SARS-CoV-2 IgG levels and neutralizing anti-*Omicron* variant activity in FHM, FNO and HW after the third dose of vaccine (T post-3D). Statistical significance is calculated by the Mann-Whitney test (*p<0.05, ****p<0.00001). Diamonds represent outliers. ns, not significant. **(C)** Data plotted are percentage values of positive subjects with cross-reactive IgG anti-spike variants (BA.4/BA.5, BF.7, BQ.1, XBB.1.5). The cut-off for serum neutralization is an inhibitory dilution used as recommended by the manufacturer. Comparisons among groups were conducted by Pearson’s Chi-square test only for T post-3D (p<0.001).

The neutralizing activity of antibodies against the *Omicron* variant of the virus (B.1.1.529) was tested at T2 and T post-3D. In the three groups, the percentage of subjects with cross-reactive IgG anti-Spike variant B.1.1.529 was higher after the third vaccine dose than at T2 (**Figure 3A**). Nevertheless, only 40% of FHM had *Omicron* neutralizing antibodies, while this activity was present in 80% of FNO and 95% of HW subjects at T post-3D. Interestingly, among FHM subjects, those with neutralizing anti-*Omicron* antibodies had significantly higher levels of anti-SARS-CoV-2 spike IgGs. The same was observed, although to a lesser extent, in FNO subjects. In contrast, no significant correlation between IgG levels and anti-*Omicron* neutralizing activity was observed in HW subjects (**Figure 3B**). The FHM *Omicron* (+) subjects were gender balanced and the median age between the + and - *Omicron* groups was virtually the same (76 vs. 77 years).

It was observed that the percentage of the subjects whose sera were able to interfere with the binding of the *Omicron* variant to ACE2 at the T2 time point was very low (**Figure 3A** black bars). To exclude that the sera at the T2 time point were unable to inhibit the binding of *Omicron* to ACE2 due to a technical artifact (i.e., old or degraded sera), we tested their ability to inhibit the binding of the *Alpha* and *Delta* variants to ACE2. As shown in **Supplementary Figure 3**, sera from HW and FNO cohorts, could inhibit the binding of the two variants to ACE2 at the T2 time point. Interestingly, they were still able to inhibit the binding even at T3 (**Supplementary Figure 3A**) and at T4 (**Supplementary Figure 3B**) time points. These results not only confirmed the integrity of the serum conservation but also demonstrated that the antibodies present in the sera after 2 vaccine doses were able to inhibit, not only the *Alpha* variant against whom the vaccine was originally designed but also the *Delta* variant. At the same time, they were unable to inhibit the *Omicron* variant.

To assess whether the neutralizing activity of the sera was also effective with other reported *Omicron* variants carrying different mutations, we tested them at T2 and T post-3D in each group against the following variants: BA.4/BA.5, BF.7, BQ.1, XBB.1.5. At T2 all subjects had no or very few neutralizing antibodies against all variants. We observed with interest that the third dose of vaccine elicits a neutralization activity depending on the cohort and *Omicron* variants. In particular, between 20% and 40% FNO subjects had neutralizing antibodies against BA.4/BA.5, BF.7, BQ.1, and XBB.1.5 variants. Notably, 100% of HW subjects had neutralizing antibodies against BF.7 variant, 50% (BA.4/BA.5), 50% (BQ.1) and 60% (XBB.1.5). Further, we observed that the FHM cohort had no or very little neutralizing activity against all variants tested at T post-3D (**Figure 3C**).

The neutralizing activity of sera to SARS-CoV-2 was also determined using a pseudovirus neutralization assay on the sera of 10 individuals from each cohort. The neutralization assay against wild type and *Omicron* pseudoviruses were tested in parallel. Dose–response curves represent the neutralizing activity of the serum of vaccinated participants against SARS-CoV-2 pseudoviruses carrying the wild type (D614G) (**Supplementary Figure 3C**) or *Omicron* spike protein (**Supplementary Figure 3D**). The results showed that, after the third dose of vaccine, all FNO and HW in the study had serum antibodies inhibiting wild type and *Omicron* variants. Conversely, antibodies inhibiting wild type virus were only in 7 FHM sera, and antibodies inhibiting the Omicron variant were in 6 FHM sera. HW had a trend to lower IC50, mainly against the Omicron variant (**Supplementary Figure 3E**). Neutralization is slightly poorer for FHM (0.2%) and FNO (0.28%) than for HW (0.5%).

**Figure 4:**
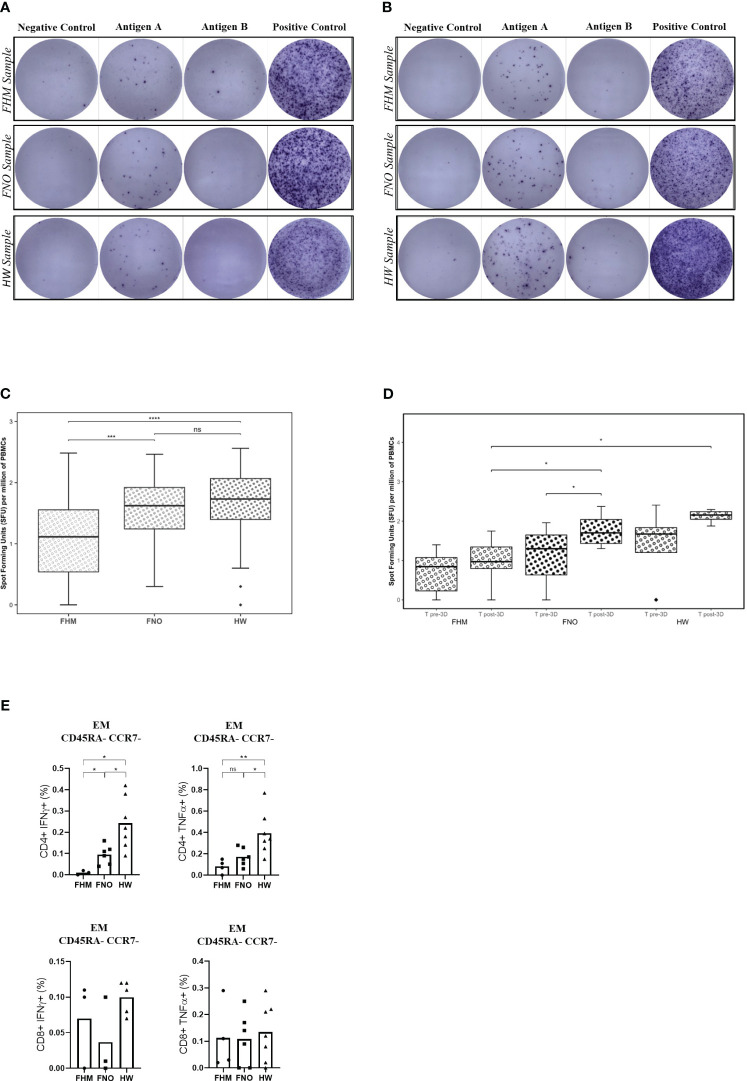
Anti-SARS-CoV-2 spike protein T-cell responses, assessed by ELISPOT. **(A)** PBMCs collected at T pre-3D time point. **(B)** PBMCs collected at T post-3D time point. Exemplary images on the digital microscope. Each sample is tested on a raw of wells; the negative control is on the left, the positive control is on the right, and the two central wells contain the sample in the study (Antigen well A and Antigen well B). **(C)** Data obtained after the third dose of the SARS-CoV-2 vaccine (381 days after the first dose of Pfizer BNT162b2). Data in the graph are expressed as spot-forming units (SFUs) per million PBMCs. The significance between pools was determined by the Kruskal-Wallis test. Diamonds represent outliers. The two-tailed Mann-Whitney test gives significance in two out of three comparisons. Precisely: ***p=0.00018 for FHM, ****p=0.00001 for the FNO and p=0.171 for the HW cohort, respectively. ns= not significant. **(D)** Comparison between T pre-3D and T post-3D for each cohort and for each time-point. Statistical significances were calculated by paired Wilcoxon test (comparisons through time-points) and by unpaired Mann-Whitney test (comparisons by groups). *p<0.05. Diamonds represent outliers. **(E)** Spike-specific T-cell responses. PBMCs of vaccinated participants (FHM, FNO, HW) were stimulated overnight with a pool of overlapping peptides covering the whole wild-type SARS-CoV-2 spike protein and analyzed by flow cytometry. Spike-specific effector memory (EM) CD8+ or CD4+ T cells producing IFN-γ or TNF-α are indicated as a fraction of total circulating T cells of the same subset. Each symbol represents an individual sample. Significance was determined using the Mann-Whitney test (*p<0.05, **p<0.01). ns, not significant.

SARS-CoV-2-specific T-cell activation after the third vaccine doseTo assess the functional T-cell response after vaccination, secretion of IFN-γ cytokine by T cells was measured at T pre-3D and T post-3D time points (**Figures 4A, B**). T cells were retrieved from PBMCs from blood samples collected at T pre-3D and T post-3D. Retrieved T cells were exposed to an antigen spike peptide pool or to phytohaemagglutinin and cell culture media alone as a positive and negative control, respectively (46, 47).

Representative images of the assay results are shown in **Figure 4A** (pre-3D) and **Figure 4B** (post-3D). Dark spots represent areas of IFN-γ secretion from T cells, indicating their activation (reactive = spot forming units [SFU] of test well – SFU of negative control ≥6 spots). Tests at post-3D (4B) show a higher number of spots compared to tests at pre-3D (4A). These latter tests had similar results to control tests on samples obtained before the vaccination campaign (**Supplementary Figure 6**). Activation of memory T cells was present in all groups, but it was 80% less in cells from FHM than in cells from FNO and HW subjects (**Figure 4C**). We performed the same analysis at T pre-3D and compared the results with those at T post-3D. As shown in **Figure 4D**, there is a trend of significance of the increase in T-cell activation in each group between the two experimental times, except in the FHM group, the less responsiveness to vaccination.

We then characterized the antigen-specific T cells through the definition of their differentiation status using CCR7 and CD45RA markers to define naïve (N), central memory (CM), effector memory (EM) and terminally differentiated effector (EMRA) populations (see **Supplementary Figure 4** for gating strategy) (37). Gating sequentially selects lymphocytes and singlets by scatter analysis following the gating for live cells, CD3+ cells, CD4+/CD8+ cells and T memory. T Central Memory (CM) are CCR7^+^CD45RA^-^; T Naïve (N) are CCR7^+^CDRA^+^; T Effector Memory (EM) are CD45RA^-^CCR7^-^; T EM CD45RA^+^ (EMRA) are CD45RA^+^CCR7^-^. The percentages of antigen-specific T cells are shown as values obtained by subtracting background values from the peptide-free control for each sample. Effector memory (EM) T-cells are defined as CD45RA-CCR7-. Effector memory CD8+ or CD4+ Spike-specific T cells were demonstrated in the PBMCs of the three cohorts after the third dose of vaccine by flow cytometry. The proportion of Spike-reactive IFN-γ- or TNF-α- producing effector memory CD4+ T cells was significantly lower in FHM than in FNO and HW (**Figure 4E**). Three of the FHM tested for T-cell activation had myeloma (only one of them had received daratumumab), and one had myeloproliferative disease.

Representative flow cytometry plots gated on CD4+ EM or CD8+ EM T cells showing production of IFNγ and TNFα following peptide pool stimulation are shown in **Supplementary Figure 5**.

### Breakthrough infections and death events after the third vaccine dose

**Figure 5:**
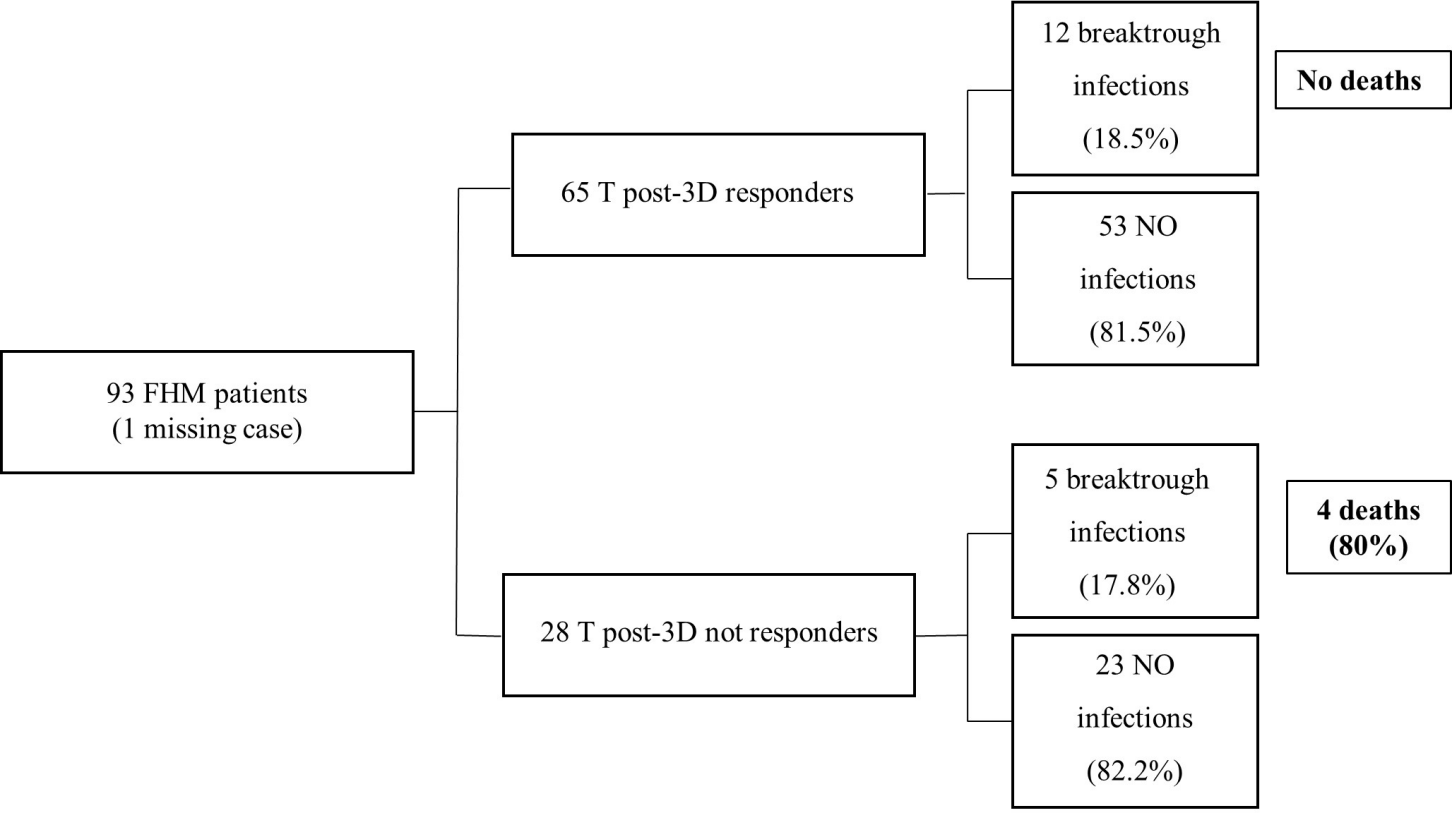
Incidence of SARS-CoV-2 infection and death in patients with hematological malignancy, according to the antibody response to the third dose of vaccine. Vaccine response of FHM subjects after the third dose of vaccine. The results are clustered in responders and not responders. The number of breakthrough infections is shown. The IgG values are calculated using LIAISON^®^ SARS-CoV-2 TrimericS IgG assay (cut-off 34 BAU/mL).

Overall, 41 subjects enrolled in the study underwent SARS-COV-2 infection between November 2021 and February 2022. Among them, 18 were FHM, 10 were FNO, and 13 were HW. The time to infection after the third dose of vaccine and the number of infected subjects were not statistically different in the three groups, but death and severe symptoms were more frequent in FHM than in the other groups (p <0.001, **Table 6**). In the group of FHM, death occurred for 3/6 infected subjects who were not responders to the third vaccine dose in terms of anti-spike antibodies (≤34 BAU/mL at a T post-3D time point, with the TrimericS IgG assay) (**Figure 5**). On the contrary, no cases of death were observed in FHM who had an antibody response to the third dose of vaccine and were infected with SARS-COV-2. No fatal events were observed in the FNO and HW cohorts as well.

**Table 6:** SARS-CoV-2 infections during the study, after the third dose of vaccine.

	FHM	FNO	HW	p-value
Time to infection T post-3D (days)				
**Median (min - max)**	101 (58 – 195)	169 (26 – 207)	139 (53 – 186)	0.255*
Breakthrough infections T post-3D				
**N (%)**	18 (19)	10 (13)	13 (20)	0.409**
Symptoms				
**N (%)**	3/18 deaths 4/18 severe symptoms 4/18 mild symptoms 7/18 no symptoms	6/10 mild symptoms 4/10 no symptoms	9/13 moderate symptoms 1/13 mild symptoms 3/13 no symptoms	<0.001**

*Kruskall-Wallis test; ** Chi-square test.

## Discussion

This observational study found that FHM responded to vaccination against SARS-COV-2 with IgG production above the protective cut-off after the second and third doses of the vaccine, although this response was lower than that observed in FNO and HW. A higher antibody level was reached after the third dose than after the second dose, both in FHM and the other groups. FHM had *Omicron*-neutralizing antibodies less frequently than FNO and HW. Specific T-cell activation after the third dose of vaccine was present in all the groups of subjects in the study, but this response, similarly to the antibody response, was lower in FHM than in FNO and HW. Remarkably, death due to COVID-19 during the study was recorded only in hematologic patients with no antibody response above the protective cut-off after the third dose of the vaccine.

Several authors have previously reported low response to the first and the second doses of the anti-SARS-COV-2 vaccine in FHM compared to HW (48, 49). After the third vaccine dose, we observed a similarly different response between FHM and HW or FNO. Indeed, our results showed that the third dose of BNT162b2 elicited a reduced immunologic response in FHM. The antibody level was increased about 10-fold 3 weeks after the third vaccine dose in FHM and 26-fold in FNO. In addition, a high proportion of FHM did not reach a protective level of IgG production.

The FHM cohort included patients affected by lymphomas, myelomas, and myeloproliferative diseases. We did not observe a different antibody response in the three patient groups. On the contrary, clustering the groups among patients treated with anti-B-cell antibodies revealed that patients on active treatments had an impaired antibody response. These results confirm previous ones reporting antibody impairment with these therapies (17).

Although we observed that the incidence of SARS-COV-2 infection was not correlated with the level of anti-SARS-CoV-2 IgG reached after the vaccination in all the groups included in the study, we recorded death only in FHM who did not reach an antibody level above the protective cut-off. This observation suggests the importance of repeated vaccine doses in FHM, as it was observed that higher antibody levels could be obtained in this population after the third dose than after the second one. No deaths due to COVID-19 occurred in infected FNO or infected and otherwise HW, independently of the level of immunologic response.

In a different setting, Aleman et al. reported that a third mRNA anti-SARS-CoV-2 vaccine dose significantly augmented cellular and humoral immune responses, including the *Omicron* variant, in patients with malignant myeloma (33). A third vaccination was associated with significantly improved neutralization capacity per antibody in a cohort of patients with hematologic cancer (20). Enssle et al. found that the serological response increased after the third dose in myeloma patients, although the neutralization capacity against *Omicron* was poor (32). These findings agree with our observations and contribute to prompt repeated vaccination of blood cancer patients.

Recently, the vaccine booster has been shown to impact B-cell clones somatic hypermutation, leading to increased affinity antibodies with neutralizing activity against divergent spike variants (50, 51). We found *Omicron*-neutralizing antibodies in a low proportion of FHM after the third dose. Nonetheless, it is possible that, besides the antibody levels, mutated antibody repertoires could be obtained by repeated doses.

We found that FHM, FNO, and HW had relevant titres of neutralizing antibodies to the *Omicron* variant only after receiving the third dose of the vaccine, which was originally designed against a different variant of the virus. It is speculated that the high level of anti-spike antibodies obtained after the third vaccine dose may protect against the infection with the *Omicron* variant of the virus.

Over the past year there have emerged vaccine-resistant SARS-CoV-2 variants of concern. To get more insight into the breadth of antibody responses, we also analyzed neutralizing capacity against BA.4/BA.5, BF.7, BQ.1, XBB.1.5 *Omicron* variants. Of note, our results indicate that the sera from the FHM cohort had no or very little neutralizing activity thus suggesting that these patients have to be subjected to the new vaccination campaign using the bivalent mRNA booster vaccine (52). On the contrary, the other two cohorts had a good neutralizing activities against the BF.7 variant, and although to a lesser extent, against the other variants too.

We are unaware of previous studies investigating T-cell response to the third dose of the SARS-CoV-2 vaccine in patients with leukemia/lymphoma. In a different setting, we confirmed a previous observation (32) that the SARS-CoV-2-specific memory T-cell response and the levels of anti-SARS-CoV-2 spike antibodies are not correlated. Indeed, the T-cell IFN-γ production in response to the third dose of the SARS-CoV-2 vaccine was lower in samples from FHM than in samples from HW and FNO. Results obtained by Enssle et al. showed that patients with malignant myeloma without a response in SARS-CoV-2-specific T-cell counts had a trend toward decreased IFN-γ production, expressed as SFUs, after variant-specific peptide stimulation; this further illustrates a reduced functional IFN-γ response in T-cell non-responders (32). We may speculate that the inconsistency of cellular and antibody response, as well as an overall reduction in both responses, in FHM, could not only be linked to impaired general conditions; it could be due to the impact of the neoplasia on the hematopoietic milieu influencing the possibility to mount cellular immune responses. In addition, antitumor therapies should play a role, as shown by Fendler et al., who found that blood cancer patients receiving B cell-depleting therapies within the 12 months before vaccination had the greatest risk of having no detectable neutralizing antibody titres (53). We observed that all patients on rituximab, but only one out 13 on daratumumab, could not reach a protective threshold of antibodies. This result suggests that B-cell depleting therapies may have heterogeneous impact on the antigen response to vaccine.

The available scientific literature has shown that the trimeric Spike protein used in the new DiaSorin test can detect the entire spectrum of the natural immune response to the virus instead of limiting detection to antibodies against single epitopes (54, 55). The antibodies produced upon vaccination are against the Wuhan isoform and we do not know whether this test would be able to detect IgG against variable of concern spike proteins, too. However, we have to take in mind that our cohorts are composed of vaccinated subjects with peptides spanning the Wuhan spike protein, not infected subjects.

We acknowledge that our results are obtained on limited cohorts; immunological responses are extremely variable and dependent on many confounding factors, so this study needs to be confirmed on a wider population. Our *in vitro* data show that antibodies to Wuhan variant present in the sera of vaccinated subjects are able to inhibit *Omicron v*ariants binding to the ACE2 receptor in healthcare and elderly subjects.Therefore, we reasoned that studying PBMC activation using peptides derived from the Wuhan strain in Wuhan-vaccinated subjects might give us a more easily interpretable data. Finally, we want to stress the relevance of our study results, despite the end of the COVID-19 pandemic. As the virus is nowadays endemic, the risk of infection is low but is not ruled out. As patients with hematologic malignancies who were infected had serious outcomes, and as death followed infection in some cases, prevention is advisable in the long-term. As repeated vaccine administration resulted in immunologic response up to a putatively protective threshold, we suggest that oncologic patients should receive anti-COVID vaccine, regularly, even when the risk of infection is low.

## Conclusion

Our results suggest that a third dose of the anti-SARS-CoV-2 vaccine may be useful in frailpatients, whether oncologic or not, and healthy subjects. Repeated vaccinations increased the antibody and T-cell response in all the cohorts. Although responses were lower in FHM, repetition of administration provided increased antibody levels and improved cellular response. These objectives appear clinically relevant as we observed that only FHM with no antibody levels above the protective cut-off died from COVID-19 infection. On the contrary, infection in subjects with a satisfactory immunologic response was associated with mild symptoms.

## Data availability statement

The raw data supporting the conclusions of this article will be made available by the authors, without undue reservation.

## Ethics statement

The studies involving humans were approved by Comitato Etico Centrale I.R.C.C.S. Lazio Sezione IRCCS I.F.O. – Fondazione G.B. Bietti. The studies were conducted in accordance with the local legislation and institutional requirements. The participants provided their written informed consent to participate in this study.

## Author contributions

FM, GP, GC, AMe and SdM contributed to the conception and design of the study. VL, CMo, LdL, CA, MC, EPr, EPi, FDM, and SdM performed the experiments. BM, IT, ES, and MPa performed statistical analysis. FPi, FCa, FPe, MPo, RP, FCar, LC, CMa, OdB, BV, AMe, AMo, EP, PF, ALM participated in the enrolment of the subjects and the collection of samples. FM, GP and SdM wrote the manuscript. All authors contributed to the article with critical reading. VL and CMo: these authors have contributed equally to this work and share the first authorship. GC and SdM: these authors have contributed equally to this work and share the last authorship. GP and SdM: these authors share the corresponding authorship. All authors contributed to the article and approved the submitted version.

## Supplementary material

Supplementary Figure 1 Response rates for anti-SARS-CoV-2 spike IgG in FHM, FNO, and HW cohorts at T pre-3D and T post-3D. Percentage of responders according to two different cut-offs: **(A)** IgG level > 15 AU/mL and **(B)** IgG level > 80 AU/mL. Pearson’s Chi-square test was used to compare groups (p<0.001 respectively).

Supplementary Figure 2 Levels of anti-SARS-CoV-2 IgG in each cohort according to gender at reported time points. No differences between genders were observed either by groups or over time. The Mann-Whitney non-parametric test was used. Diamonds represent outliers.

Supplementary Figure 3 Neutralizing antibodies. **(A)** Levels of neutralizing antibodies for the anti-*Alpha* spike variant in healthcare workers (HW) and fragile not oncologic patients (FNO) at T2 and T3. **(B)** Levels of neutralizing antibodies for the anti-*Delta* spike variant in HW and FNO at T2 and T4. Statistical significance was calculated by the Mann-Whitney test. The neutralizing activity of sera was determined using a pseudovirus neutralization assay **(C, D)**. Dose–response curve represents the neutralizing activity of the serum of vaccinated participants (FHM, FNO, HW) against SARS-CoV-2 pseudovirus carrying the wild type (D614G) **(C)** or *Omicron* spike protein **(D)**. **(E)** Samples with < 50% inhibition at 10% serum were excluded from the IC50 calculation (2/10 FHM against D614G viral pseudoparticles; 4/10 FHM against *Omicron* viral pseudoparticles). Outlier detection was performed with the ROUT test using GraphPad Prism. Significance was determined using the Mann-Whitney test.

Supplementary Figure 4 Gating strategy for the identification of T cell subsets. Gating sequentially selects for lymphocytes by scatter analysis, for singlets, for live cells, for CD3+ cells, for CD4+/CD8+ cells, and for T memory. T Central Memory (CM) are CCR7+CD45RA-, T Naïve (N) are CCR7+CDRA+, T Effector Memory (EM) are CD45RA-CCR7-, T EM CD45RA+ (EMRA) are CD45RA+CCR7-.

Supplementary Figure 5 Spike-specific T cell responses characterized by cytokine production. Representative flow cytometry plots gated on CD4+ EM (top) or CD8+ EM (bottom) T cells showing the production of IFNγ and TNFα following peptide pool stimulation. Numbers in gates indicate percentages of positive cells.

Supplementary Figure 6 PBMCs collected at T0 time point. Exemplary images on the digital microscope. Each sample is tested on a raw of wells; the negative control is on the left, the positive control is on the right, and the two central wells contain the sample in the study (Antigen well A and Antigen well B).
