# Supplementary material for: Dynamics of humoral and cellular response to three doses of anti-SARS-CoV-2 BNT162b2 vaccine in patients with hematological malignancies and older subjects
Source: Front Immunol. 2024 Jan 26;14:1221587. doi: 10.3389/fimmu.2023.1221587 (PMC10853639; doi:10.3389/fimmu.2023.1221587)
Supplement: Supplementary file 1 [file DataSheet_1.docx]

**SUPPLEMENTARY MATERIAL**

**METHODS**

**1. Serum isolation and serological studies**

For serum isolation, whole blood samples in BD Vacutainer® SST™ tubes (BD, Italy) were clotted at room temperature for at least 1 hour and then centrifuged for 10 min at 800 g. Serum samples were stored at +4 °C until use, then aliquoted and stored at -80°C for SARS-COV-2 antibody detection and future use.

Levels of IgG antibodies against S1/S2 antigens of SARS-CoV-2 were measured with the LIAISON^®^ SARS-CoV-2 S1/S2 IgG commercial test (Diasorin, Italy). The LIAISON^®^ SARS-CoV-2 S1/S2 IgG test is a quantitative chemiluminescent immunoassay (CLIA), fully automated on the LIAISON^®^ XL platform, for the detection of IgG antibodies against the subunits S1 and S2 of the SARS-CoV-2 spike protein. According to the manufacturers’ technical manual, the result of a LIAISON^®^ SARS-CoV-2 S1/S2 IgG test has been reported as positive with a signal of 15 AU/mL or higher, equivocal between 12 and 15 AU/mL and negative <12 AU/mL. The cut-off to discriminate positive samples was based on a concordance level of 94.4% between the LIAISON^®^ IgG titer of 15 AU/mL and the Plaque Reduction Neutralization Test 90% (PRNT90) at 1:40 ratio. At a LIAISON^®^ value of 80 AU/mL, a concordance of 100% with a 1:160 PRNT90 titer was observed. To date, PRNT90 is a gold standard for evaluating the relative concentrations of virus-specific neutralizing antibodies. The robustness of the concordance evidence between this CLIA and the PRNT90 allowed for LIAISON^®^ SARS-CoV-2 S1/S2 IgG by DiaSorin^®^ to be approved by the FDA in April 2020 (1).

In order to facilitate the serological method standardizations and allow data-set comparisons and harmonization across laboratories, the first WHO International Standard and the first WHO Reference Panel of anti-SARS-CoV-2 immunoglobulin have been established (2). As a consequence, from September 2021, serological studies were performed by using the LIAISON® SARS-CoV-2 TrimericS IgG assay (Emergency Use Authorization, EUA). This indirect CLIA technology detects IgG antibodies to SARS-CoV-2 in human serum and plasma samples. The principal components of the test are paramagnetic particles (solid phase) coated with recombinant trimeric SARS-CoV-2 spike protein and a conjugate reagent containing an anti-human IgG mouse monoclonal antibody linked to isoluminol derivatives. The assay is intended to assess the presence of antibodies to SARS-CoV-2, including neutralizing antibodies, in human serum or plasma. The positive percent agreement of the LIAISON^®^ SARS-CoV-2 TrimericS IgG test compared with polymerase chain reaction (PCR) for samples ≥15 days after diagnosis was determined to be 98.7% (95% CI: 94.5–99.6%) according to the manufacturer’s instruction for use; conversely, the negative percent agreement was 99.5% (95% CI: 99.0–99.7%) in 1899 blood donor samples collected prior to COVID-19 outbreak. The antibodies in the tested samples were quantified in arbitrary units per milliliter (AU/mL) and converted to BAU/mL according to the manufacturers’ information regarding the WHO standard. The conversion to BAU was AU/mL×2.6 (3).

**2. Binding Neutralization Assay *Alpha* variant**

Neutralizing anti-receptor-binding domain (RBD) antibodies were measured by a COVID-19 Spike-ACE2 Binding Assay Kit (Raybiotech Life, Inc., GA, USA). This test detects semi-quantitative antibodies against the SARS-CoV-2 spike protein in human serum. Specifically, serially diluted serum samples and provided controls were incubated with recombinant SARS-CoV-2 spike RBD protein, pre-coated on the 96-well plates. Subsequently, the wells were washed and incubated with substrate and stop solution as suggested by the manufacturer. Finally, the absorbance of the sample, after the coloring reaction, was measured at 450 nm using a UV/Vis microplate spectrophotometer. The presence of neutralizing antibodies in the serum was assessed as a percentage of binding inhibition, which was calculated using the following formula: % Binding inhibition = [(OD of “0 mM” positive control – OD of Test Reagent)/OD of “0 mM” positive control)] × 100.

***Delta* and *Omicron* variant**

To test functional neutralizing antibodies against the *Delta* (B.1.617.2) and *Omicron* variants of SARS-CoV-2, two commercially available ELISA kits were used: SARS-CoV-2 Neutralizing Antibodies Detection Kit (B.1.617.2 *Delta* variant), Cat. No. AG-48B-0007-KI01, Adipogen and SARS-CoV-2 Neutralizing Antibody Titer Serologic Assay Kit (B.1.1.529 *Omicron* variant), ACROBiosystems. Both assays identify the presence or absence of specific neutralizing antibodies by testing the interaction of the RBD of the SARS-CoV-2 spike protein and the recombinant human ACE2 receptor (hACE2). Specifically, the Adipogen kit for *Delta* variant measurement contains 96-well microplates. Wells are coated with recombinant SARS-CoV-2 spike RBD protein, and human ACE2 protein coupled to horseradish peroxidase (HRP) is added to detect the lack of binding and the presence of competitive antibodies. In contrast, the ACROBiosystems kit provides pre-coated plates of hACE2 and uses HRP-conjugated SARS-CoV-2 spike RBD protein for antibody recognition. Both assays were performed according to the manufacturing instructions. After the coloring reaction, the absorbance of the samples (inversely proportional to the presence of SARS-CoV-2 neutralizing antibodies) was measured at 450nm using a UV/Vis microplate spectrophotometer. The percentage of neutralization was calculated as follows: (1-(OD of the sample)/OD of negative control) × 100. The Adipogen kit defines a cut-off value of 20%, while for the ACROBiosystems kit, the cut-off value corresponds to 32%. The inhibition percentage of ≥20% or ≥32% indicates the presence of neutralizing antibodies against the SARS-CoV-2 *Delta* or *Omicron* variant, respectively.

***Omicron* variants *(BA.4 / BA.5 / BF.7 / BQ.1 / XBB.1.5)***The Gene Tex SARS-CoV-2 Neutralizing Antibody ELISA Kit (Omicron BA.4/BA.5/BF. 7/ BQ.1 /XBB.1.5) Cat. No. GTX538288, is an in vitro assay for qualitative SARS-CoV-2 neutralizing antibody (Nab) screening. According to the continuous evolution of SARS-CoV-2, the kit includes recombinant His-tagged SARS-CoV-2 Spike RBD proteins for the Omicron variant BA.4 I BA.5, Omicron variant BF. 7, Omicron variant BQ.1, and Omicron variant XBB.1.5, for Omicron variant-specific neutralization analysis. The assay employs a recombinant ACE2 protein immobilized in the wells of the included 96-well plate (12 x 8-well strips). Control antibody (either NAb or Non-NAb) mixtures or samples are pipetted into the appropriate wells, followed by addition of the recombinant His-tagged spike RBD protein(s). Neutralizing agents in the samples (or in the NAb Control Antibody solution) react with RBD proteins to prevent them from binding to ACE2. After washing away unbound proteins and antibodies, an HRP-conjugated anti-His tag antibody is added to bind to the immobilized recombinant RBD proteins. The wells are washed again to remove free conjugated antibody, and TMB (3,3' ,5,5'-tetramethylbenzidine) substrate is added for color development. The Stop Solution changes the color from blue to yellow, and the intensity of the color (measured at 450 nm) inversely correlates with the titer of the anti-RBD protein neutralizing antibodies (Inhibition Rate (IR) = [1 - (average absorbance value of tested sample/ average absorbance value of NC)] x 100%. The inhibition percentage of ≥30% indicates the presence of neutralizing antibodies against the SARS-CoV-2 *Omicron* variants.

**3. Assessment of activated T effector cells**

**Isolation of PBMCs**

PBMCs were isolated from “buffy coats” (whole blood concentrates without serum) via density gradient centrifugation using Lympholyte^®^-H Cell Separation Media (CEDARLANE^®^). Briefly, heparinized blood was diluted with phosphate-buffered saline (PBS) and layered on top of Lympholyte in SepMate tubes (Stemcell). After completing the centrifugation (1800 rpm, 40 minutes, no brake), the mononuclear cells from the plasma/Lympholyte interface were collected using a plastic Pasteur pipette and then washed twice with PBS (1400 rpm, 10 minutes). Cells were counted using a hemacytometer with trypan blue solution (Lonza) to determine cell viability. Isolated PBMCs were cryopreserved in fetal bovine serum (EuroClone) containing 10% dimethyl sulfoxide (DMSO; Sigma-Aldrich) and stored in liquid nitrogen.

**Activation of effector T-cells**The T-SPOT COVID-19 test was used. An enzyme-linked immunospot (ELISPOT) assay identifies T cells in PBMCs that release IFN-γ in response to stimulation with SARS-CoV-2 spike S1 and/or nucleocapsid peptides. It is a commercialized, standardized ELISPOT platform that allows reproducible measurements of T cells reactive to SARS-CoV-2 antigens. The T-SPOT COVID-19 test builds on the T-SPOT platform (Oxford Immunotec, a PerkinElmer Company). To quantify the activation of effector T cells, we used the T-SPOT COVID-19 test. As part of the T-cell response, SARS-CoV-2 antigens on the vaccine could activate both CD4+ and CD8+ effector T cells, which, if further stimulated by these antigens, produce IFN-γ cytokine. The T-SPOT COVID-19 test includes over 253 SARS-CoV-2 peptides (15-mer peptides overlapping by 11 amino acids) in two antigen peptide pools; one pool contains peptides from the spike S1 protein, including the RBD, and the other contains peptides from the nucleocapsid protein. PBMCs previously cryopreserved were thawed in warm complete RPMI medium, washed, and counted on Guava^®^ easyCyte Flow Cytometer (Oxford Immunotec, a PerkinElmer Company). Then, 1.000.000 cells/well were plated into four wells of a 96-well plate. The antigen peptide pools were added to the wells coated with anti-IFN-γ antibodies. The T-cell mitogen phytohemagglutinin was added to the positive control wells, and cell culture media alone to the negative control wells. Once plated, the microtiters were incubated in a humidified CO_2_ incubator set at 37°C, 5% CO_2_. After 16–20 hours, the wells were washed with PBS and stained using a conjugated secondary antibody (1 hour at 2–8° C) bound to any IFN-γ captured on the membrane. After washing to remove unbound IFN-γ, the substrate was added for 7 minutes at room temperature to produce dark spots of insoluble precipitates indicating areas of IFN-γ secretion from T cells. The spot-forming units (SFUs) were counted using an automated ELISPOT plate reader (CTL, Shaker Heights, OH, USA). Results were ‘invalid’ if the negative control had more than 10 SFUs or the positive control had fewer than 20 SFUs when the antigen wells were non-reactive. Results were ‘reactive’ when the SFUs in the higher of the two antigen wells minus the negative control were ≥6, ‘non-reactive’ when the SFUs in both antigen wells minus the negative control were ≤6, and ‘borderline’ when the SFUs in the higher of the antigen wells minus the negative control were 5, 6 or 7.

**4. In vitro stimulation of PBMCs**

PBMCs were thawed and rested for 4 hours in 10% FBS-supplemented RPMI-1640. Then, 2×106 PBMCs were seeded in U-bottom 96-well plates in a total volume of 200 μl and stimulated for 12–16 hours at 37 °C in 10% FBS-supplemented RPMI-1640 with PepTivator^®^ SARS-CoV-2 protein S peptide pool (Miltenyi Biotec, cat. 130-127-953) or CEFX Ultra SuperStim Pool (JPT, PM-CEFX-2) at the final concentration of 1 μg/mL of each peptide or with an equal volume of water, in the presence of Brefeldin A (Thermo Fisher Scientific, cat. 00-4506-51), as previously described (4).

**5. Flow cytometry staining and acquisition**

Cells were stained for viability (LIVE/DEAD™ Fixable Violet Dead Cell Stain, Thermo Fisher Scientific, cat. L34955). Afterward, cells were incubated with Human Fc Block (BD, cat. 564220) followed by staining for surface markers using the following antibodies: CD3 (UCHT1) FITC, 1:200 (Thermo Fisher Scientific, cat. 11-0038-42), CD4 (RPA-T4) PerCP-eFluor 710, 1:100 (Thermo Fisher Scientific, cat. 46-0049-42), CCR7 (CD197) APC, 1:100 (BD Biosciences, cat. 566762), CD8 (SK1) APC-eFluor 780, 1:200 (Thermo Fisher Scientific, cat. 47-0087-42), CD45RA (HI100) BV605, 1:100 (BioLegend, cat. 304134) in FACS buffer for 20 minutes at 4 °C. Afterward, samples were fixed and permeabilized using Cytofix/Cytoperm (BD Biosciences, cat. 554722) for 20 minutes at 4 °C. Intracellular staining was performed in Perm/Wash buffer (BD Biosciences, cat. 554723) for 30 minutes at 4 °C with the following antibodies: IFNγ (4S.B3) PE, 1:100 (Thermo Fisher Scientific, cat. 12-7319-42), TNFα (MAb11) PC7, 1:100 (Thermo Fisher Scientific, cat. 25-7349-82). The stained samples were acquired through a CytoFLEX ﬂow cytometer (Beckman Coulter), and the data were analyzed using CytExpert software (Beckman Coulter). Cytokine expression in the presence of only water (with no peptides) was considered background and subtracted from the values measured with stimuli.

We focused on IFNγ and TNFα since they are two crucial players in antiviral and vaccine-induced responses. These two cytokines were assessed by intracellular cytokine staining (ICS) following stimulation overnight with the peptide pools, using an in-house validated flow cytometry antibody panel for Th1 response (5).

Phenotype analysis of antigen-specific T cells was only performed in subjects with at least 10 cells detected in the respective cytokine-positive gate.

**6.** **Production of SARS-CoV-2 pseudoparticles**

To produce the pseudotyped lentiviral particles, 8.5×106 HEK-293T cells were plated in a 15 cm dish in complete DMEM medium and co-transfected using Lipofectamine 2000 Transfection Reagent (Thermo Fisher Scientific) on the following day with 35 µg of pLenti CMV Puro LUC (w168-1) (Addgene #17477), 26.3 µg of pCMVR8.74 (Addgene #22036) and 26.3 µg of SARS-CoV-2 D614G-19-aa Ctrunc-Spike plasmid or SARS-CoV-2 Omicron-19-aa Ctrunc-Spike plasmid. Then, 12 hours after transfection, the medium was replaced with a 5% FBS-supplemented DMEM medium; 48 hours after transfection, the supernatant was collected and clariﬁed by centrifugation for 10 minutes at 1500 rpm. Viral pseudoparticle suspensions were aliquoted and stored at -80 °C.

We did not calculate viral particles. We rely on the luminescence signal, as explained below. A luciferase assay with Bright-Glo Luciferase Assay System (Promega) was used to determine viral titer. Serial dilutions of the virus-containing supernatant were applied to infect HEK293T-hACE2 and an amount of virus-containing supernatant where there was sufficient signal (>200-fold) above cells-only background was selected for the neutralization assay.

**7.** **Neutralization assay with pseudotyped particles**

For neutralization assay with pseudotyped particles, HEK293T-hACE2 cells (8.5 x10^6^ cells were seeded) were transduced with SARS-CoV-2 pseudovirus previously incubated with a serial 2.5-fold dilution of heat-inactivated serum to obtain an 8-point dose-response curve. Luciferase assay was performed using Bright-Glo™ Luciferase System (Promega, cat. E2620) and Victor plate reader. Measured relative light units (RLUs) were normalized to controls, and dose–response curves were generated using GraphPad Prism software, as previously described (6).

SARS-CoV-2 pseudoparticles were tested for their ability to specifically infect susceptible HEK293T-hACE2 and not HEK293T-WT target cells. A luciferase assay with Bright-Glo Luciferase Assay System (Promega) was used to determine viral titer. Serial dilutions of the virus-containing supernatant were applied to infect HEK293T-hACE2 and an amount of virus-containing supernatant where there was sufficient (>200-fold) signal above cells-only background was selected for the neutralization assay. In addition, no neutralizing serum and a neutralizing monoclonal antibody were used as a negative and positive control of neutralization, respectively.

**References**

1. Bonelli F, Sarasini A, Zierold C, Calleri M, Bonetti A, Vismara C, et al. Clinical and analytical performance of an automated serological test that identifies S1/S2-neutralizing IgG in COVID-19 patients semiquantitatively. J Clin Microbiol (2020) 58:e01224-20. doi: 10.1128/JCM.01224-20.
2. WHO. Standards of vaccines for coronavirus disease (COVID-19). <https://www.who.int/news-room/feature-stories/detail/standardization-of-vaccines-for-coronavirus-disease-covid-19>.
3. Lukaszuk K, Kiewisz J, Rozanska K, Dabrowska M, Podolak A, Jakiel G, et al. Usefulness of IVD kits for the assessment of SARS-CoV-2 antibodies to evaluate the humoral response to vaccination. Vaccines (Basel) (2021) 9:840. doi: 10.3390/vaccines9080840.
4. Salvatori E, Lione L, Compagnone M, Pinto E, Conforti A, Ciliberto G, et al. Neoantigen cancer vaccine augments anti-CTLA-4 efficacy. NPJ Vaccines (2022) 7:15. doi: 10.1038/s41541-022-00433-9.
5. Jingyou Yu,1, Lisa H. Tostanoski, Lauren Peter, Noe B. Mercado, Katherine McMahan, et al. DNA vaccine protection against SARS-CoV-2 in rhesus macaques. Science (2020) 369(6505): 806–811. doi: [10.1126/science.abc6284](https://doi.org/10.1126%2Fscience.abc6284)
6. Compagnone M, Pinto E, Salvatori E, Lione L, Conforti A, Marchese S, et al. DNA-vaccine-induced immune response correlates with lower viral SARS-CoV-2 titers in a ferret model. Vaccines (Basel) (2022) 10:1178. doi: 10.3390/vaccines10081178.
