# Supplementary figures and images for: Dynamics of humoral and cellular response to three doses of anti-SARS-CoV-2 BNT162b2 vaccine in patients with hematological malignancies and older subjects
Source: Front Immunol. 2024 Jan 26;14:1221587. doi: 10.3389/fimmu.2023.1221587 (PMC10853639; doi:10.3389/fimmu.2023.1221587)

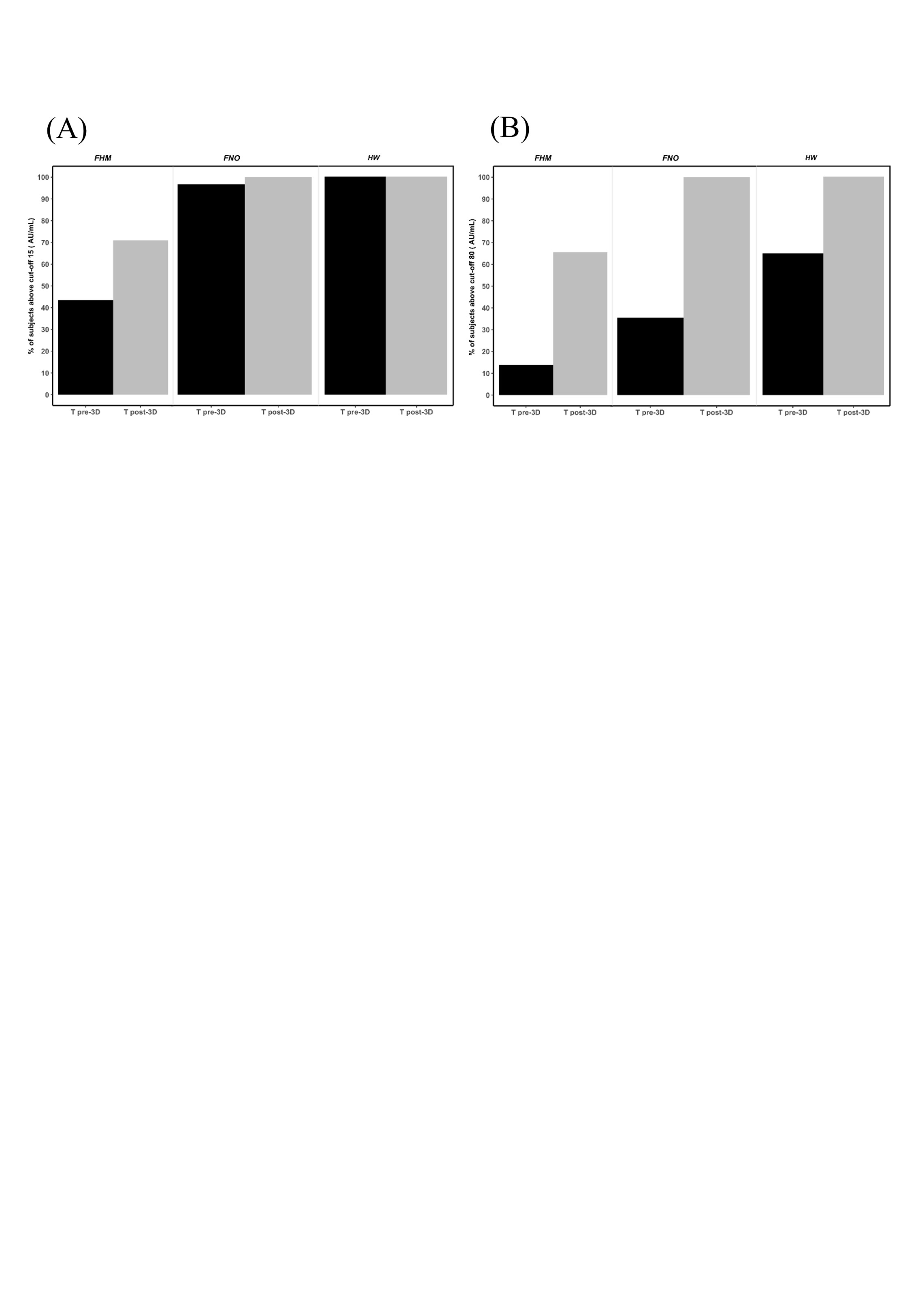

Supplement: Supplementary Figure 1 — Response rates for anti-SARS-CoV-2 spike IgG in FHM, FNO, and HW cohorts at T pre-3D and T post-3D. Percentage of responders according to two different cut-offs: (A) IgG level > 15 AU/mL and (B) IgG level > 80 AU/mL. Pearson’s Chi-square test was used to compare groups (p<0.001 respectively). [file Image_1.jpeg]

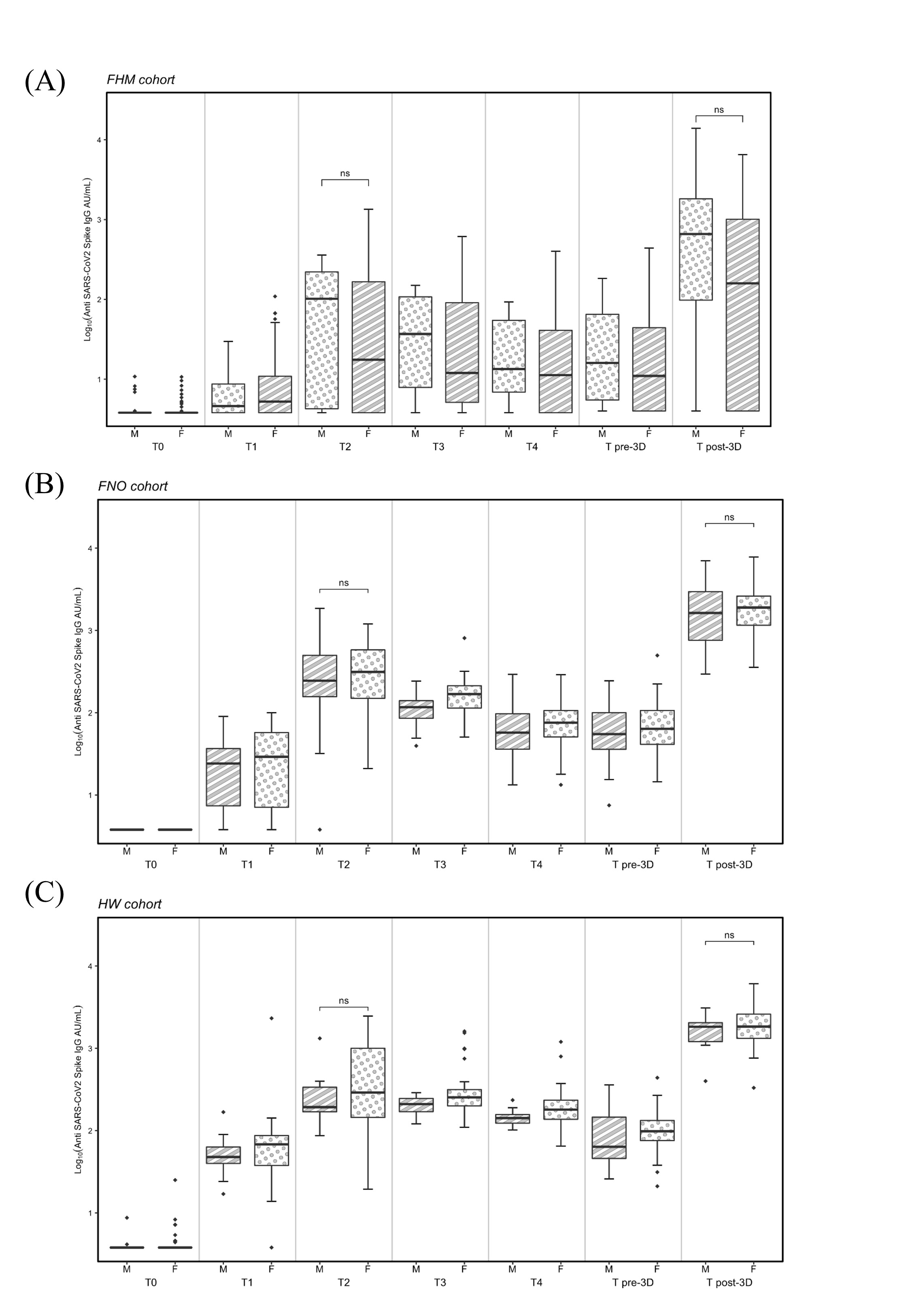

Supplement: Supplementary Figure 2 — Levels of anti-SARS-CoV-2 IgG in each cohort according to gender at reported time points. No differences between genders were observed either by groups or over time. The Mann-Whitney non-parametric test was used. Diamonds represent outliers. [file Image_2.jpeg]

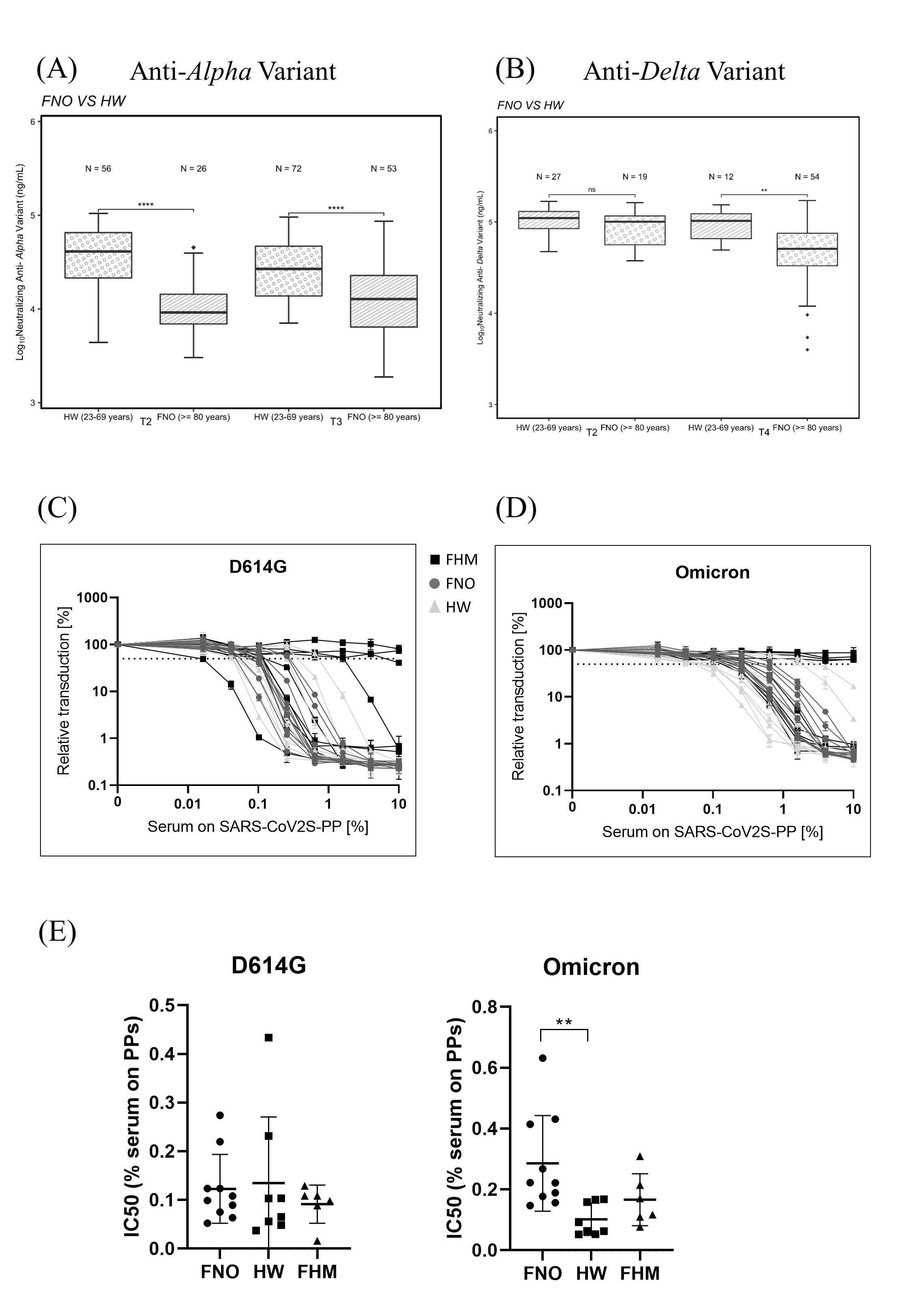

Supplement: Supplementary Figure 3 — Neutralizing antibodies. (A) Levels of neutralizing antibodies for the anti-Alpha spike variant in healthcare workers (HW) and fragile not oncologic patients (FNO) at T2 and T3. (B) Levels of neutralizing antibodies for the anti-Delta spike variant in HW and FNO at T2 and T4. Statistical significance was calculated by the Mann-Whitney test. The neutralizing activity of sera was determined using a pseudovirus neutralization assay (C, D). Dose–response curve represents the neutralizing activity of the serum of vaccinated participants (FHM, FNO, HW) against SARS-CoV-2 pseudovirus carrying the wild type (D614G) (C) or Omicron spike protein (D). (E) Samples with < 50% inhibition at 10% serum were excluded from the IC50 calculation (2/10 FHM against D614G viral pseudoparticles; 4/10 FHM against Omicron viral pseudoparticles). Outlier detection was performed with the ROUT test using GraphPad Prism. Significance was determined using the Mann-Whitney test. [file Image_3.jpeg]

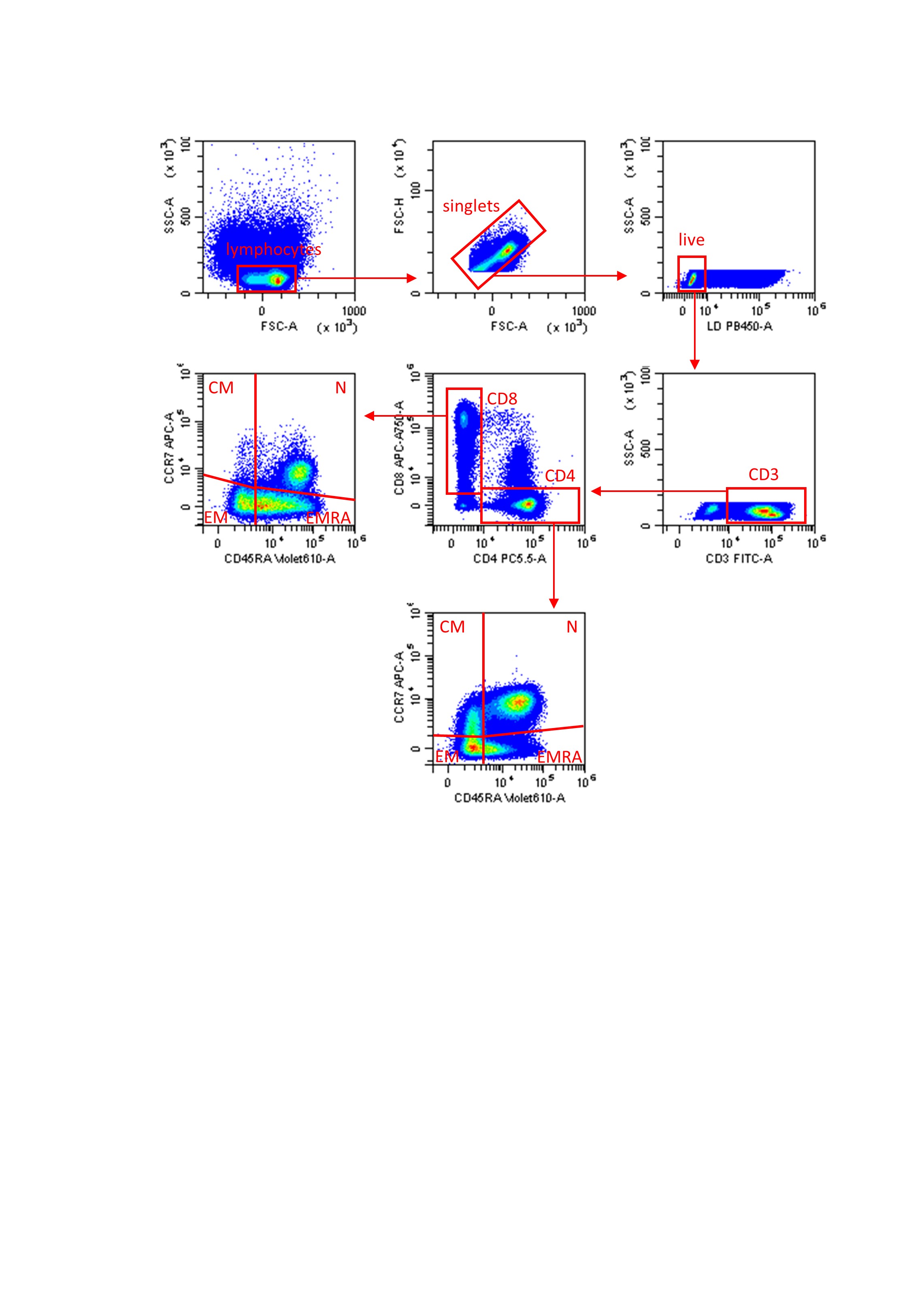

Supplement: Supplementary Figure 4 — Gating strategy for the identification of T cell subsets. Gating sequentially selects for lymphocytes by scatter analysis, for singlets, for live cells, for CD3+ cells, for CD4+/CD8+ cells, and for T memory. T Central Memory (CM) are CCR7+CD45RA-, T Naïve (N) are CCR7+CDRA+, T Effector Memory (EM) are CD45RA-CCR7-, T EM CD45RA+ (EMRA) are CD45RA+CCR7-. [file Image_4.jpeg]

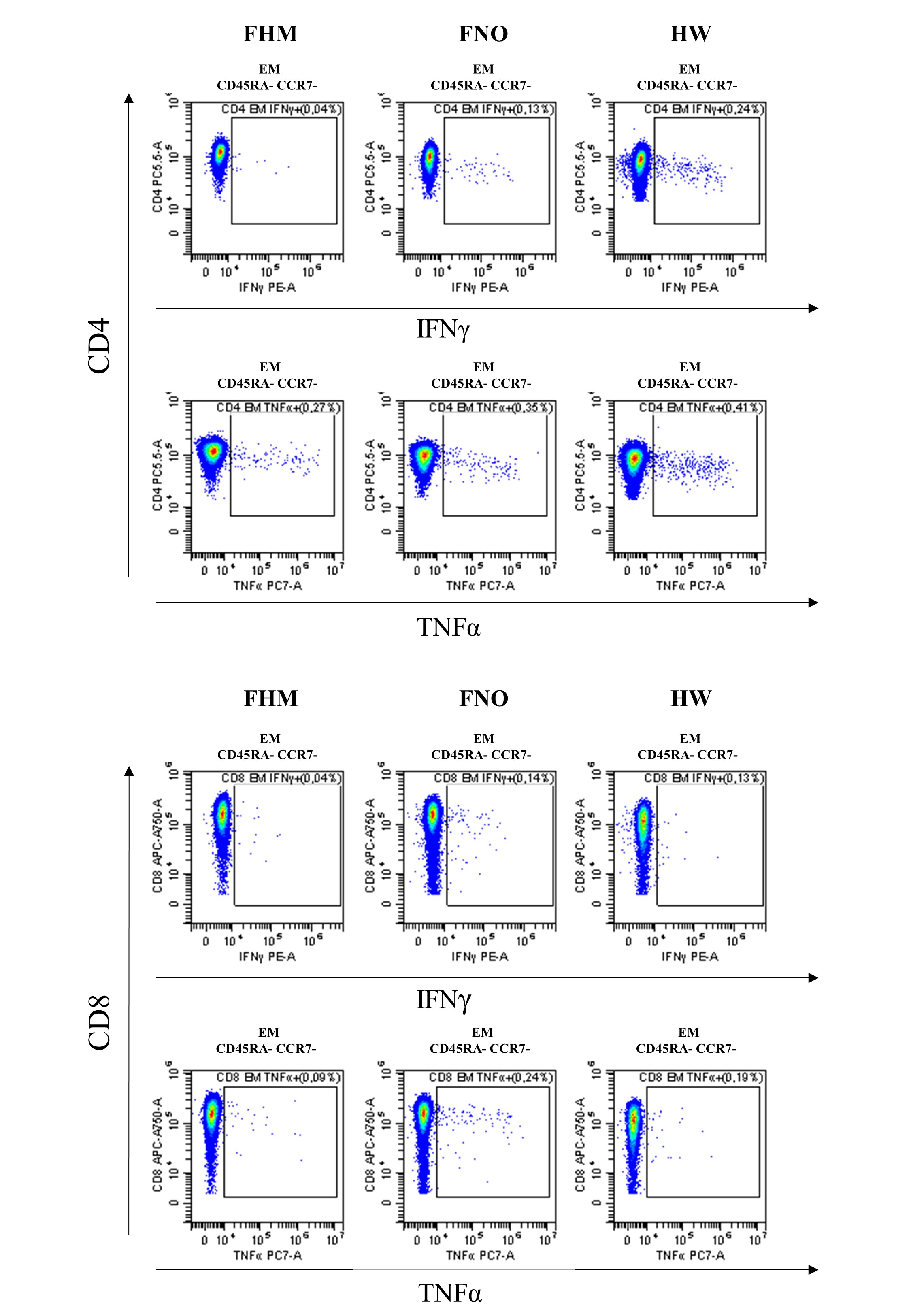

Supplement: Supplementary Figure 5 — Spike-specific T cell responses characterized by cytokine production. Representative flow cytometry plots gated on CD4+ EM (top) or CD8+ EM (bottom) T cells showing the production of IFNγ and TNFα following peptide pool stimulation. Numbers in gates indicate percentages of positive cells. [file Image_5.jpeg]

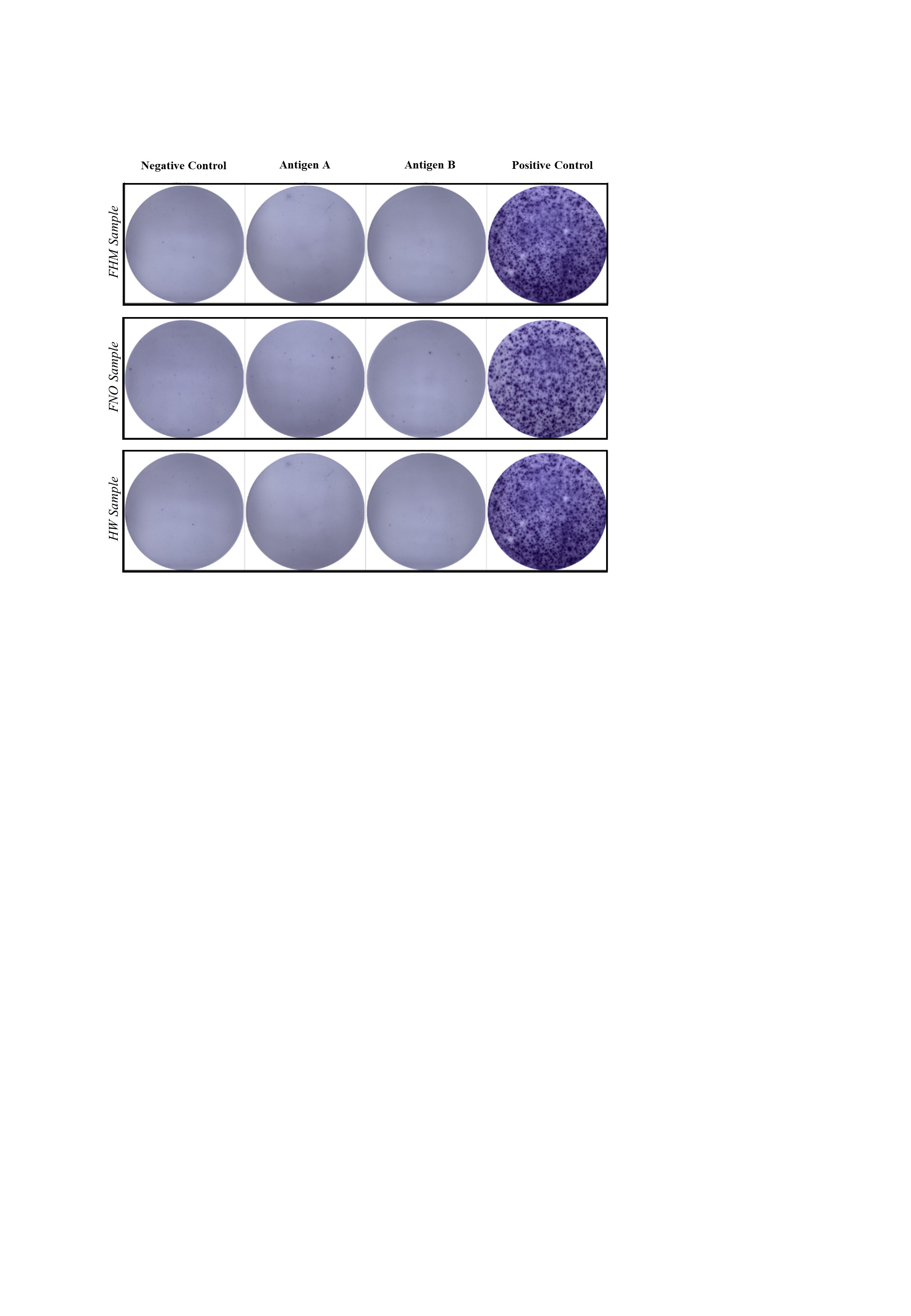

Supplement: Supplementary Figure 6 — PBMCs collected at T0 time point. Exemplary images on the digital microscope. Each sample is tested on a raw of wells; the negative control is on the left, the positive control is on the right, and the two central wells contain the sample in the study (Antigen well A and Antigen well B). [file Image_6.jpeg]
